# Comparison of Polarized Versus Other Types of Endurance Training Intensity Distribution on Athletes’ Endurance Performance: A Systematic Review with Meta-analysis

**DOI:** 10.1007/s40279-024-02034-z

**Published:** 2024-05-08

**Authors:** Pedro Silva Oliveira, Giorjines Boppre, Hélder Fonseca

**Affiliations:** 1 Faculty of Sport, Research Centre in Physical Activity, Health and Leisure (CIAFEL), University of Porto, Rua Dr. Plácido Costa, 91, 4200-450 Porto, Portugal; 2 Laboratory for Integrative and Translational Research in Population Health (ITR), Porto, Portugal; 3 Nucleus of Research in Human Movement Science, University Adventista, 3780000 Chillan, Chile

## Abstract

**Background:**

Polarized training intensity distribution (POL) was recently suggested to be superior to other training intensity distribution (TID) regimens for endurance performance improvement.

**Objective:**

We aimed to systematically review and meta-analyze evidence comparing POL to other TIDs on endurance performance.

**Methods:**

PRISMA guidelines were followed. The protocol was registered at PROSPERO (CRD42022365117). PubMed, Scopus, and Web of Science were searched up to 20 October 2022 for studies in adults and young adults for ≥ 4 weeks comparing POL with other TID interventions regarding* V*O_2_peak, time-trial (TT), time to exhaustion (TTE) or speed or power at the second ventilatory or lactate threshold (V/P at VT_2_/LT_2_). Risk of bias was assessed with RoB-2 and ROBINS-I. Certainty of evidence was assessed with GRADE. Results were analyzed by random effects meta-analysis using standardized mean differences.

**Results:**

Seventeen studies met the inclusion criteria (*n* = 437 subjects). Pooled effect estimates suggest POL superiority for improving *V*O_2_peak (SMD = 0.24 [95% CI 0.01, 0.48]; *z* = 2.02 (*p* = 0.040); 11 studies, *n* = 284; *I*^2^ = 0%; high certainty of evidence). Superiority, however, only occurred in shorter interventions (< 12 weeks) (SMD = 0.40 [95% CI 0.08, 0.71; *z* = 2.49 (*p* = 0.01); *n* = 163; I^2^ = 0%) and for highly trained athletes (SMD = 0.46 [95% CI 0.10, 0.82]; *z* = 2.51 (*p* = 0.01); *n* = 125; *I*^2^ = 0%). The remaining endurance performance surrogates were similarly affected by POL and other TIDs: TT (SMD = – 0.01 [95% CI -0.28, 0.25]; *z* =  − 0.10 (*p* = 0.92); *n* = 221; *I*^2^ = 0%), TTE (SMD = 0.30 [95% CI – 0.20, 0.79]; *z* = 1.18 (*p* = 0.24); *n* = 66; *I*^2^ = 0%) and V/P VT_2_/LT_2_ (SMD = 0.04 [95% CI -0.21, 0.29]; *z* = 0.32 (*p* = 0.75); *n* = 253; *I*^2^ = 0%). Risk of bias for randomized controlled trials was rated as of some concern and for non-randomized controlled trials as low risk of bias (two studies) and some concerns (one study).

**Conclusions:**

POL is superior to other TIDs for improving * V*O_2_peak, particularly in shorter duration interventions and highly trained athletes. However, the effect of POL was similar to that of other TIDs on the remaining surrogates of endurance performance. The results suggest that POL more effectively improves aerobic power but is similar to other TIDs for improving aerobic capacity.

**Supplementary Information:**

The online version contains supplementary material available at 10.1007/s40279-024-02034-z.

## Key Points

**Table:** 

Polarized training is superior to other training intensity distribution models for the improvement of * V*O_2_peak. There was no evidence of polarized training superiority for any of the remaining endurance performance surrogates investigated.
Polarized training superiority was mostly evident for interventions lasting less than 12 weeks. When exercise interventions were longer than 12 weeks, * V*O_2_peak was shown to increase similarly in those using POL or other training intensity distribution models.
Baseline endurance performance level was shown to influence the effect of polarized training on * V*O_2_peak improvement in highly trained/national level athletes.

## Introduction

Endurance performance is highly dependent on variables such as volume, frequency, intensity, and training intensity distribution (TID). Since high-level endurance athletes perform high training volumes, close to a maximum physiologically tolerable limit [1], adequate manipulation of TID is fundamental for performance optimization [2, 3]. TID can be characterized according to the percentage of training volume spent on zones demarcated by established physiological thresholds. Three [4] or five [5] intensity zones are usually defined. The most used model defines Zone 1 (Z1) as intensity below the first ventilatory or lactate threshold, Zone 3 (Z3) as above the second ventilatory or lactate threshold, and Zone 2 (Z2) between Z1 and Z3 [6, 7].

Of the most frequently used TIDs [4, 7] polarized training (POL) and threshold training (THR) seem to be the most effective in endurance performance improvement [8–10]. POL consists of high training volumes in Z1 (75–80%), moderate volumes in Z3 (15–20%), and a small fraction in Z2 (< 10%). In THR training, volume in Z2 is emphasized (> 35%), with the remaining volume distributed between Z1 and Z3 [11]. THR was, until recently, the predominant endurance training model [12–15]. However, recent evidence suggesting the superiority of POL has led to its preferential adoption [16] by high-level [7] and recreational athletes [17] alike. Preferred adoption of THR until recently was based on the argument that training mostly between ventilatory thresholds optimally recruited aerobic metabolism [18, 19]. The rationale supporting POL superiority is based on knowledge of the signaling pathways involved in mitochondrial biogenesis [20]. The intracellular calcium signaling pathway is mainly potentiated by high training volumes at low intensity [21], while the 5' AMP-activated protein kinase (AMPK) pathway is optimally activated by depleting the cell ATP, particularly during high-intensity efforts [22, 23]. A combination of both low and high training intensity would, therefore, optimally recruit these signaling pathways, enhancing endurance performance [18].

Despite these physiological arguments, studies comparing POL with other TIDs have provided conflicting results, with some showing superiority [8, 24–27] and others not [28–30]. Conflicting findings might be due to the retrospective nature and reliance on training diaries in several studies [7], poor control of training variables [11, 31], small sample sizes [2, 29, 30, 32], and low statistical power. A previous systematic review with meta-analysis has addressed this issue [33], but the number of studies available at that time was still limited. Thus, uncertainty remains as to the most appropriate training model for optimizing endurance performance. Due to the growing interest in POL, several studies have been recently published. This increase justifies the need to perform a new systematic review with meta-analysis of the available evidence to address this issue.

The aim of this study was to determine if POL training is superior to other TIDs for the improvement of endurance performance. To test this hypothesis, a set of variables correlated with endurance performance was selected, namely (i) maximum oxygen consumption (*V*O_2_peak), (ii) time-trial (TT), iii) time to exhaustion (TTE), and (iv) velocity or power at second ventilatory or lactate threshold (V/P at VT_2_/LT_2_) [7]. VO_2_peak is the highest O_2_ consumption attained during an incremental exercise [34], and anaerobic threshold corresponds to the intensity above which workloads induce exponential increases in blood lactate concentration, and therefore it is not possible to sustain a steady-state condition [35]. TT is the time necessary to complete a given distance and is frequently used as an endurance performance test in specific distances [36], while TTE is commonly used as a measure of exercise capacity or tolerance since it requires maintaining a fixed workload for the longest duration possible [37]. These variables are well correlated with endurance performance [38–40] in several athletic backgrounds, and are frequently used as surrogates or predictors of endurance performance by both researchers and coaches alike.

## Methods

This systematic review with meta-analysis followed the PRISMA (Preferred Reporting Items for Systematic Reviews and Meta-analyses) 2020 guidelines (Appendix S1 of the Electronic Supplementary Material (ESM)) [41]. The protocol was defined and registered at PROSPERO (CRD42022365117) prior to beginning data collection and analysis.

### Eligibility Criteria

Only studies published in scientific peer-reviewed journals in English were considered for the analysis. There were no restrictions regarding publication date. Eligibility criteria for study selection were followed the PICOS (Participants, Intervention, Comparator, Outcome, Study design) framework:

(i) Participants: Humans of both sexes; age between 15 and 65 years; absence of comorbidities or physical limitations that could hinder exercise participation without restrictions at the onset of the intervention.
(ii) Intervention: Endurance training interventions following the POL intensity distribution principle [11] with a frequency of three or more sessions/week and ≥ 4 weeks of intervention. There were no restrictions regarding the settings in which the intervention took place (e.g., elite, professional or recreational context).
(iii) Comparator: Endurance training interventions following other TID principles such as, but not limited to, THR, pyramidal training (PYR), high-intensity interval training (HIIT) or sprint interval training (SIT), high-volume training (HVT) performed for three or more sessions per week, ≥ 4 weeks of intervention.
(iv) Outcome: The outcomes of interest were surrogates of endurance performance. To be included in the analysis, studies should include at least one of the following outcomes: (a) VO_2_peak; (b) TT; (c) TTE, or (d) V/P at VT_2_/LT_2_.
(v) Study design: Randomized controlled trials or non-randomized controlled trials with at least two groups, one experimental and one comparator, and at least a baseline and a post-intervention measurement.

### Information Sources and Search Strategy

PubMed, Scopus, and Web of Science databases were used to perform the searches, which were carried out between 10 and 20 October 2022. No filters were applied during searches. An example of the specific search strategy conducted in PubMed was as follows: (("polarized training") OR ("polarized endurance training")) AND (((((("training intensity distribution") OR ("endurance training")) OR ("pyramidal training")) OR ("threshold training")) OR ("high intensity training")) OR ("high volume low intensity training")). A preprint of the search strategies from PubMed, Web of Science and Scopus is presented in Appendix S2 of the ESM. Reference lists of the included studies were also screened for potentially relevant studies (snowball technique). Whenever information regarding relevant variables was missing, the corresponding author was contacted by email and ResearchGate® to request the missing information.

### Study Selection

After the initial database searches, references were downloaded to an EndNoteTM 20 for Mac (ClarivateTM) database for automated removal of duplicates followed by manual inspection and removal of remaining duplicates. Subsequently, titles were screened and studies that included polarized training interventions in humans were selected. After this stage, abstracts of the selected studies were reviewed and all of those potentially meeting the inclusion criteria were selected and full-text analysis was performed to ascertain inclusion of the study according to the pre-established criteria. Two researchers (PO and GB) independently conducted the literature search and study selection and then compared their results to ensure accuracy. Disagreements were resolved by consensus and included a third researcher (HF).

### Data Collection Process

PO and GB independently collected the mean and standard deviation of all data items of interest following a pre-defined data extraction sheet including information regarding authors, publication date, study design, sample size, TID, exercise intervention type, duration/volume, frequency, outcomes, and results of relevant outcomes for the POL group and other TIDs group. Data collected were subsequently compared between researchers for consistency assessment. Cases of ambiguity regarding data collection were solved by consensus including a third researcher (HF). No automation tools were used for data extraction. Whenever the necessary data were not available in the text or tables, the WebPlotDigitizer tool was used to extract the information from plots. Several studies included more than one intervention group of interest for the analysis. Whenever these cases were identified, results were included as separate reports in the analysis and identified with superscript letters indicating different sporting modalities (i.e., cycling, swimming, running) or different TIDs (THR, HIIT, HVT).

### Data Items

Variables relevant for assessing the superiority or inferiority of POL compared to other TIDs for endurance performance improvement were collected and variables related to the study implementation context and exercise intervention characteristics were collected. The primary outcome was peak oxygen uptake (*V*O_2_peak; continuous variable) measured by indirect calorimetry. Secondary outcomes were: (i) time to complete a pre-specified distance (TT; continuous variable); (ii) TTE in a pre-specified maximal exercise testing protocol (continuous variable); and (iii) external load (velocity/power) at which the VT_2_/LT_2_ occurs (continuous variable). Due to the ambiguity regarding this concept in the literature [42], we assumed as synonymous concepts lactate threshold (LT), lactate turn point (LTP), and respiratory compensation threshold. Additional variables assessed were:

(i) Variables related to the description of the exercise intervention: weekly training volume, duration, distance, frequency, and weekly or total training impulse (TRIMP) [43].

(ii) Variables related to the description of the participants: sport, competitive level [44], years of practice, number of subjects per group, age and sex.

(iii) Other variables: study design, study implementation location (e.g., country), and funding sources. There was a need to convert time to exhaustion to percent variation change between post- and pre-intervention since this was the metric used in some studies.

### Study Risk of Bias Assessment

The Cochrane risk of bias tools for randomized controlled trials (RoB-2) [45] and non-randomized studies of interventions (ROBINS-I) [46] were used to assess the risk of bias of individual studies. Bias assessment that involved RoB-2 and ROBINS-I domains were rated as having low risk, some concerns, or high risk. RoB-2 is divided into five dimensions of bias: (D1) arising from the randomization process, (D2) due to deviations from intended intervention, (D3) due to missing outcomes data, (D4) in measurement of the outcome, and (D5) in selection of the reported results. ROBINS-I is divided into seven dimensions of bias: (D1) due to confounding, (D2) due to participants selection, (D3) in classification of interventions, (D4) due to deviations from intended interventions, (D5) due to missing data, (D6) in measurement of outcomes, and (D7) in selection of the reported results. PO and GB independently completed the risk-of-bias analysis, which was later reviewed by a third author (HF). Where inconsistencies emerged, the original articles were re-analyzed until a consensus was reached.

### Effect Measures

For determining the superiority of POL in comparison to other TIDs for endurance performance improvement, the effect size of individual studies was calculated as mean difference or standardized mean difference (SMD) between intervention and comparator groups. The SMD was calculated as the difference between the mean of the POL group and comparator group divided by the pooled SD, and was employed for analysis of TT and V/P at VT_2_/LT_2_ since these outcomes were reported by different measurement units in different studies. Mean difference was used for VO_2_peak and TTE. Effect measures from meta-analysis were determined through a random-effects inverse variance model using SMD (Hedges’ *g*) with 95% CI [47]. Overall effect (*Z*-test) was considered statistically significant at *p* < 0.05.

### Synthesis Methods

A qualitative synthesis of the included study’s findings structured around the different exercise endurance training protocols, for example, pyramidal training (PYR), threshold training (THR), high-volume low intensity training (HVLIT), high intensity training (HIT) in comparison with POL interventions, was conducted. A random effects meta-analysis using the inverse variance method [48] was also performed to compare the effect of POL with other TIDs regarding the primary and secondary outcome measures, and results were displayed by forest plots. Fourteen of the 17 studies included in the systematic review were included in the meta-analysis. Sub-analyses were also performed to determine if the comparison between POL and other TIDs differed by sex (males vs. females), intervention duration (interventions ≤ 12 weeks’ vs. ≥ 12 weeks’ duration), starting endurance performance level (highly trained/national level vs trained/developmental) and TT duration (< 12 min or ≥ 12 min). For TT duration, a cut-off of 12 min was chosen, based on the usual *V*O_2_max test duration, and considering that shorter efforts are related more to *V*O_2_peak and longer ones to V/P at VT_2_/LT_2_.

A sensitivity analysis was also performed by excluding one study at a time to determine the consistency of the results. To perform these analyses, the “meta” package in *R* software was used. The *Z*-test was used to assess overall effect and was considered statistically significant at *p* < 0.05. The *I*^2^ statistic was used to assess between-studies heterogeneity and was qualitatively characterized as: 0–40% not important, 30–60% moderate, 50–90% substantial, and 75–100% considerable. A visual inspection of the funnel plot and the Egger's linear regression method test were used to assess publication bias in two variables (*V*O_2_peak and V/P at VT_2_/LT_2_) in cases where at least ten studies were available [49].

### Certainty of Evidence Assessment

We followed the Grading of Recommendations Assessment, Development, and Evaluation (GRADE) tool (online version: Copyright © 2021, McMaster University and Evidence Prime Inc. All rights reserved) to determine the certainty of the evidence of the findings regarding the study primary outcome (*V*O_2_peak) [50, 51]. Risk of bias, inconsistency, indirectness of the evidence, imprecision, and publication bias are the key domains used in the evaluation of the certainty of the evidence by GRADE and are graded as high, moderate, low, or extremely low. Certainty of evidence of the included studies in this systematic review with meta-analysis was evaluated in terms of having adequate sample size, narrow confidence intervals, and absence of heterogeneity. GRADE was independently assessed by two authors (PO and GB), with disagreements managed by consensus or through a third author (HF).

## Results

### Study Selection

**Fig. 1:**
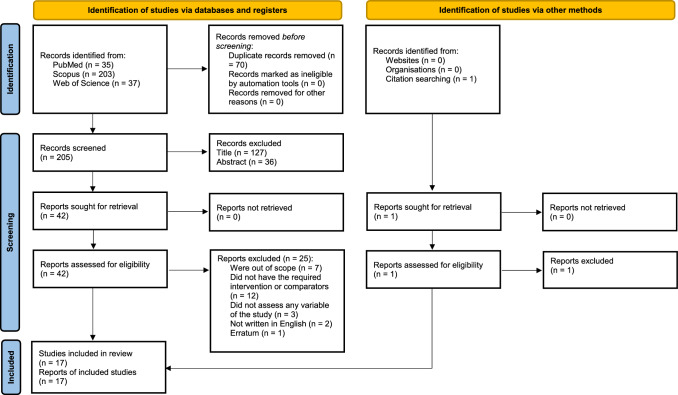
PRISMA 2020 flow diagram

The flowchart details the studies included in the review. An initial search returned 275 results (35 PubMed, 203 Scopus, and 37 Web of Science). After removing duplicates, 205 reports remained. The screening of titles and abstracts for eligibility criteria resulted in the exclusion of 163 reports, leaving 42 articles for full text analysis. Of these, 25 were excluded for not meeting the eligibility criteria: seven were outside the research objective, 12 did not had the necessary intervention or comparators, three did not assess any of the variables of interest, two were not written in English, and one was an erratum. Snowballing revealed one additional potentially suitable article. After screening the abstract, a full text analysis was performed revealing that this article did not meet the eligibility criteria. Seventeen studies were finally included in the systematic review. There were no discrepancies between raters in the selection of studies to be included in the final analysis (Fig. 1).

### Characteristics of the Studies Included

Table 1 summarizes all the characteristics of the 17 studies included in this review that compared the effect of an intervention based on POL with other TIDs on improving endurance performance. The total number of participants included was *n* = 437 (317 males, 89 females, 31 undisclosed), of which 183 participated in POL interventions and 223 in other TIDs. One study (31 participants) did not indicate the number of subjects in each group [52]. Most studies (*n* = 15) included only or predominantly men. Only one study included only or predominantly women [53], and only one study included a balanced sample of both sexes [54]. All participants in the selected studies were adults or young adults, ranging in age from 17 ± 3 [55] to 44.2 ± 14.6 years [24]. The sample size in each study varied between *n* = 11 [26] and *n* = 52 participants [53], with a median of *n* = 22 per study.

**Table 1:** Summary of the selected studies

Study	Sample characteristics			Competitive level	TID % (Z1/Z2/Z3)	Training load	Training characteristics	Outcomes used^a^	Main findings
	Sex	Age, y	*V*O_2_max baseline						
Stöggl et al. (2014) [8] RCT (9 weeks)	POL: 12 ♂ HIIT: 10 ♂ HVT: 11 ♂ THR: 8 ♂	31 ± 6	62.6 ± 7.1 (range: 52–75)	Highly Trained/ National Level Endurance athletes (cross-country skiing, cycling, triathlon, middle or long-distance running)	POL: 68 ± 12/ 6 ± 8/26 ± 7 HIIT: 43 ± 1/ 0/ 57 ± 1, THR: 46 ± 7.54/7/0 HVT: 83 ± 6 / 16 ± 6/1 ± 1	NR	Total training time (h): POL: 104 ± 20 HIIT: 66 ± 1 THR: 84 ± 7 HVT: 102 ± 11 Total number of sessions (n): POL: 54 ± 3 HIIT: 47 ± 1 THR: 49 ± 3 HVT: 58 ± 3	*V*O_2_peak (mL·min^−1^·kg^−1^) TTE (s) V/P LT_2_ (km·h^−1^ or W) [Blood lactate]	*V*O_2_peak: ↑ TTE: ↑ V/P LT_2_: ↑
Muñoz et al. (2014) [17] RCT (10 week﻿s)	POL: 16 ♂ THR: 16 ♂	POL: 34 ± 9 THR: 34 ± 7	POL: 61.0 ± 8.4 THR: 64.1 ± 7.3	Trained/ Developmental endurance runners	POL: 75/5/20 THR: 46/35/19	TRIMP/week: POL: 330 ± 67 THR: 370 ± 98 Total TRIMP: POL: 3299 ± 670 THR: 3691 ± 982	Total running time (h): POL: 39.1 ± 7.9 THR: 36.3 ± 8.1 Weekly running distance averaged (km): 50 Weekly training frequency (n): 5–6	10 km TT (min)	TT: ← →
Carnes et al. (2018) [24] RCT (12 weeks)	POL: 9, 8 ♂ CFE: 12, 7 ♂	POL: 44.2 ± 14.6 CFE: 41 ± 12.9	POL: 45.9 ± 7.1 CFE: 46.5 ± 6.9	Trained/ Developmental Distance runners	POL: 85/5/10 CFE: 48/8/44	TRIMP/week: POL: 389 ± 101 CFE: 222 ± 68 Total TRIMP: POL: 4610 ± 620 CFE: 2641 ± 270	Weekly average training time (min): POL: 283 ± 75.9 CFE: 117 ± 32.2 Weekly average distance (km): POL: 47.3 ± 11.6 CFE: 19.3 ± 7.17	*V*O_2_max (mL·min^−1^·kg^−1^) 5km run TT (min)	*V*O_2_max: ↑ 5km run TT: ← →
Hebisz et al. (2021) [25] RCT (8 weeks)	POL: 10, 7♂ BT: 10, 7 ♂	POL: 18.5.0 ± 1.9 BT: 18.4 ± 1.6	POL: 57.2 ± 5.8 BT: 60.0 ± 4.8	Highly Trained/ National Level Mountain bike cyclists	NR	NR	Total training sessions (n): POL: 12 × SIT, 12 × HIIT, 16 × LIT BT: 11 × SIT, 11 × HIIT, 18 × LIT	*V*O_2_max (mL·min^−1^·kg^−1^) P VT_2_ [W] PETCO_2_, VE/VCO_2_	*V*O_2_max: ↑ P VT_2_: ← →
Neal et al. (2013) [26] RCT (6 weeks)	POL: 11♂ THR: 11 ♂	37.6	NR	Trained/ Developmental Cyclists	POL: 80/0/20 THR: 57/43/0	TRIMP/week: POL: 517 ± 90 THR: 633 ± 119	Weekly training time (min): POL: 381 ± 85 THR: 458 ± 120	40 km cycling TT(s) TTE 95% PPO (s) P LT_2_ (W) [Blood lactate]	TT: ← → TTE: ↑ P LT_2:_ ↑
Schumann et al. (2017) [27] N-RCT (12 weeks)	POL: 14 ♂ TL: 10 ♂	POL: 34 ± 7 TL: 34 ± 7	POL: 46.4 ± 5.9 TL: 47.3 ± 3.5	Trained/ Developmental Endurance runners	NR	TRIMP/week: Week 0–4: POL: 301 ± 35 TL: 285 ± 28 Week 5–8: POL: 315 ± 41 TL: 306 ± 42 Week 9–12: POL: 358 ± 52 TL: 269 ± 85	Weekly training time (h): POL: 10.8 ± 2.4 CON: 10.0 ± 2.7	*V*O_2_max (mL·min^−1^·kg^−1^) 1000m TT (min) TTE (min)	*V*O_2_max: ↑ TT: ← → TTE: ← →
Festa et al. (2020) [28] RCT (8 weeks)	POL: 19, 15 ♂ THR: 19, 16 ♂	POL: 43.2 ± 8.4 THR: 39.4 ± 8.5	POL 53.0 ± 5.9 THR: 53.7 ± 1.9	Trained/ Developmental runners	POL: 77/3/20 THR: 40/50/10	TRIMP/week: POL: 308 ± 47.46 THR: 319.8 ± 28.1 Total TRIMP: POL: 2464 ± 124 THR: 2558.2 ± 10.9	Total running time (h): POL: 29.9 ± 3.1 THR: 24.8 ± 2.0	*V*O_2_max (mL·min^−1^·kg^−1^) 2km TT (s) vRCT (km h^−1^) VE/VCO_2_	*V*O_2_max: ← → TT: ← → V RCT: ← →
Röhrken et al. (2020) [29] RCT (6 weeks)	POL: 7, 5 ♂ THR: 8, 6 ♂	POL: 29.1 ± 7.6 THR: 30.3 ± 6.1	NR	Trained/ Developmental Triathletes	POL: 75.2 ± 14.4/11.1 ± 10.9/13.7 ± 4.1 THR: 77.8 11.9/20.3 ± 10.8/2.0 ± 1.5	TRIMP/week: POL:882.0 ± 155 THR:739.0 ± 162	Weekly training time (h): POL: 10.8 ± 2.4 THR: 10.0 ± 2.7	V LT_2_ (km·h^−1^) [Blood lactate] P LT_2_ (W) [Blood lactate]	V LT_2_: ← → P LT_2_: ← →
Treff et al. (2017) [30] N-RCT (11 weeks)	POL: 7 ♂ PYR: 7 ♂	POL: 21 ± 2 PYR: 19 ± 1	POL: 68 ± 7 PYR: 64 ± 3	Highly Trained/ National Level Rowers	POL: 93/2,1 ± 1/6 ± 3 PYR: 94 ± 3/3 ± 2/2 ± 1	NR	Total rowing distance (km): POL: 1334 ± 67 PYR: 1255 ± 264 Total rowing time (min): POL: 5953 ± 315 PYR: 5919 ± 1216 Total number of sessions (n): POL: 80 ± 4 PYR: 84 ± 13	*V*O_2_max (mL·min^−1^·kg^−1^) 2000m TT (s) P LT_2_ (W) [Blood lactate]	*V*O_2_max: ← → TT: ← → P LT_2_ ↓
Selles-Perez et al. (2019) [32] N-RCT (13 weeks)	POL: 6 ♂ PYR: 7 ♂	POL: 28.5 ± 7.7 PYR 29.2 ± 6.8	Run POL: 52.8 ± 4.1 Run PYR: 58.1 ± 3.9 Bike POL: 50.5 ± 2.9 Bike PYR: 54.1 ± 5.1	Trained/ Developmental Triathletes	POL: 84.5/4.2/11.3 PYR: 77.9/18.8/ 3.3	ECOs/week: POL: 785.2 ± 244.9 PYR: 751.6 ± 234.9	Total training time (h): 155 Weekly average training time (h): POL: 11.9 ± 3.5 PYR: 11.9 ± 3.6 Total training sessions (n): PYR: 106 (28 for swimming, 34 for cycling and 44 for running) POL: (28 for swimming, 34 for cycling and 45 for running)	*V*O_2_max running (mL·min^−1^·kg^−1^) *V*O_2_max cycling (mL·min^−1^·kg^−1^) V/P VT_2_ (Running, Cycling and Swimming) PETCO_2_, VE/VCO_2_	*V*O_2_max running: ↑ *V*O_2_max cycling: ← → VT_2_ Running: ↑ VT_2_ Cycling: ← → VT_2_ Swimming: ↓
Stöggl et al. (2017) [52] RCT (9 weeks)	31	31 ± 6	61.9 ± 8.0 (range 54–75)	Highly Trained/ National Level Endurance athletes (cross-country skiing, cycling, triathlon, middle- or long-distance running)	POL: 68 ± 12/ 6 ± 7/26 ± 7 HIIT: 43 ± 1/0/ 57 ± 1 HVLIT: 64 ± 20/35 ± 21/ 1 ± 1	NR	Total training time (h): POL: 104 ± 21 HIIT: 66 ± 1 HVLIT: 93 ± 13 Total number of sessions (n): POL: 54 ± 7 HIIT: 47 ± 1 HVLT: 54 ± 8	V/P VT_2/_LT_2_ (km·h^−1^ or W) [Blood lactate]	V/P at VT_2/_LT_2_: ← →
Zapata-Lamana et al. (2018) [53] RCT (12 weeks)	POL: 14 ♀ MICT: 14 ♀ HIIT: 14 ♀ CG: 10 ♀	POL: 21.8 ± 1.9 MICT: 21.3 ± 1.4 HIIT: 21.2 ± 1.4 CG: 22.7 ± 3.2	POL: 24.5 ± 2.5 MICT: 22.7 ± 3.1 HIIT: 25.3 ± 2.6 CG: 25.0 ± 4.0	Sedentary	POL: 70–80/0/ 20–30 NR	NR	Total number of cycling sessions (n): 36 Weekly number of cycling sessions (n): 3 Weekly cycling time (min): POL: 120 MICT: 135–150 HIIT: 156	VO_2_peak (mL·min^−1^·kg^−1^)	VO_2_peak: ↑
Pla et al. (2019) [54] RCT (6 weeks)	POL: 9 THR: 13 12 ♂, 10 ♀	POL: 17 ± 3 THR: 17 ± 3	POL: 56.0 ± 11.3 THR: 56.4 ± 12.4	Highly Trained/ National Level junior swimmers	POL: 81/4/15 THR: 25/65/10	NR	Weekly training distance (km) POL: 42 ± 4 THR: 42 ± 4	100m TT (s) V LT_2_ (m/s) [Blood lactate]	TT: ↑ V LT_2_ ↓
Schneeweiss et al. (2022) [55] RCT (4 weeks)	POL: 10, 8 ♂ LIT: 8, 6 ♂	POL: 18.4 ± 4.7 LIT: 17.4 ± 1.9	NR	Highly Trained/ National Level XCO athletes	POL: 86.6/0/13.4 LIT: 100/0/0	NR	Total training time (h): POL: 25 LIT: 40	TT (300s) P LT_2_ (W) [Blood lactate]	TT: ← → P LT_2_: ← →
Filipas et al. (2022) [56] RCT (16 weeks)	POL: 14 ♂ PYR: 14 ♂	POL: 38 ± 5 PYR: 35 ± 6	POL: 69 ± 3 PYR: 68 ± 4	Trained/ Developmental runners	POL: 78–81/4–7/14–15 PYR: 77–78/ 15–17/6–7	TRIMP/week: Week 1–8: PYR: 463 ± 77 POL: 464 ± 81 Week 9–18: PYR: 462 ± 78 POL: 465 ± 79	Weekly training time (min): Week 1–8: PYR: 358 ± 63 POL: 348 ± 57 Week 9–18: PYR: 358 ± 63 POL: 348 ± 56	*V*O_2_max (mL·min^−1^·kg^−1^) 5-km TT (s) V LT_2_ (km·h^−1)^ [Blood lactate]	*V*O_2_max: ← → TT: ← → V LT_2_: ← →
Pérez et al. (2019) [57] RCT (12 weeks)	POL: 11 ♂ THR: 9 ♂	POL: 40.6 ± 9.7 THR: 36.8 ± 9.2	POL: 55.8 ± 4.9 THR 57.1 ± 5.2	Trained/ Developmental Ultra-endurance runners	POL: 79.8 ± 2.1/ 3.9 ± 1.9/ 16.4 ± 1.5 THR: 67.2 ± 4.6/33.8 ± 4.6/0	Total TRIMP: POL: 5906.0 ± 708.8 THR: 6061.2 ± 1726.2	Weekly training time (h): POL: 6.0 ± 0.8 THR: 6.5 ± 1.4 Weekly training frequency (n): 5	*V*O_2_max (mL·min^−1^·kg^−1^) TTE (s) V VT_2_ (km h^−1^) PETCO_2_, VE/VCO_2_	*V*O_2_max: ← → TTE: ↑ V VT_2:_ ← →
Hebisz et al. (2021) [58] RCT (9 weeks)	POL: 14 ♂ CG: 12 ♂	POL: 21.7 ± 7.7 CON: 20.5 ± 5.5	POL: 62.3 ± 6.4 CON: 59.6 ± 8.4	Highly Trained/ National Level Mountain bike cyclists	NR	NR	Total training sessions (n): POL: 2 × SIT, 1 × HIIT, and 2 × ET CG: 2 × HIIT, 3 × ET	*V*O_2_max (mL·min^−1^·kg^−1^)	*V*O_2_max: ↑

*BT* block training, *CFE*^*©*^ CrossFit endurance training ^©^, *CG* Control Group, *EC*O*s* Objective Load Equivalents, *ET* endurance training, *h* hours, *HIIT* high-intensity interval training, *HVLIT* high-volume low-intensity training, *km.h*^*−1*^ km per hour, *LIT* low-intensity training, *m.s*^*−1*^ m per second, *MICT* moderate intensity continuous training, *n* number of sessions, *N-RCT* Non Randomized Controlled Trial, *NR* Not reported, *P LT*_2_—power at second lactate threshold, *P VT*_2_power at second ventilatory threshold, *PETC*O_2_ end-tidal carbon dioxide pressure, *P*O*L* polarized training, *PP*O peak power output, *PYR* pyramidal training, *RCT* Randomized Controlled Trial, *RCT* respiratory compensation threshold, *s* seconds, *SIT* sprint interval training, *THR* threshold training, *TID* training intensity distribution, *TL* training load guided, *TRIMP* training impulse, *TT* time trial, *TTE* time-to-exhaustion, *V LT*_2_velocity at 2nd lactate threshold, *V RCT* Velocity at respiratory compensation threshold, *V VT*_2_velocity at 2nd ventilatory threshold, *VE/VC*O_2_ minute ventilation/carbon dioxide production, *V/P at VT*_2_*/LT*_2_ velocity or power at second ventilatory or lactate threshold, *V*O_2_max maximal oxygen uptake, *V*O_2_*peak* peak oxygen uptake, *W* Watts, *Z1* training zone 1, *Z*_2_ training zone 2, *Z3* training zone 3

^a^The main outcomes gathered from each of the studies included in the review are listed together with the method used to determine V/P at VT_2_/LT_2_

Note: Age is expressed as mean ± SD

Symbols: Female (♀), male (♂), decreased (↓), increased (↑) and equal (← →) regarding POL compared to other TIDs

The studies selected for analysis included a wide variety of endurance sports, with running and cycling being predominant. Six studies evaluated endurance runners [17, 24, 27, 28, 56], in one case ultra-endurance runners [57]. Four studies evaluated cyclists, of which one evaluated road cyclists [26], one cross-country [55], and two mountain bikers [25, 58]. Six studies evaluated other sports, namely one study of swimmers [54], one of rowers [30], two of triathletes [29, 32], and two included multisport participants, namely cross-country skiers, cyclists, medium and long-distance triathletes, and runners [8, 52]. One study included previously untrained subjects [53].

Seven studies were performed with highly trained athletes/national level athletes, of which two included mountain bikers [25, 58], one cross country cyclists [55], one swimmers [54], one rowers [30], and two a mixed sample of various disciplines [8, 52]. Nine studies evaluated trained/developmental athletes, of which six included runners [17, 24, 27, 28, 56], one of these ultra-endurance runners [57], one road cyclists [26], and two triathletes [29, 32]. One study included previously sedentary subjects [53].

The duration of training interventions ranged from 4 [55] to 16 weeks [56], with a median of 10 weeks. Three studies did not report the TID [25, 27, 58]. One study [53] reported the TID only in the POL group.

In two studies [25, 58] the training duration and the number of sessions dedicated to high- and low-intensity training were reported without mentioning the percentage of the total training time distribution attributed to each intensity. Eight studies reported the weekly TRIMP, with studies from Carnes et al. [24] and Röhrken et al. [29] showing the lowest (POL: 389 ± 101; CrossFit Endurance^©^: 222 ± 68) and highest (POL: 882.0 ± 155; THR: 739.0 ± 162) weekly value. The minimum weekly training frequency was three sessions [24] and the maximum was ten sessions [55].

VO_2_peak was assessed in 11 studies [8, 24, 25, 27, 28, 30, 32, 53, 56–58], TT in nine studies [17, 24, 26–28, 30, 54–56], TTE in four studies [8, 26, 27, 57], and V/P at VT_2_/LT_2_ in 12 studies [8, 25, 26, 28–30, 32, 52, 54–57].

### Qualitative Synthesis of Findings

For VO_2_peak, differences between groups after the intervention were not significant in six studies [28, 30, 32, 53, 56, 57]. Five studies [8, 24, 25, 27, 58] concluded that POL was superior compared to other TIDs and no studies suggested that POL was inferior. For TT, eight studies [17, 24, 26–28, 30, 55, 56] did not show significant differences between interventions, and one study [54] showed favorable results for POL. Of the four studies analyzing TTE, three reported superiority for POL [8, 26, 57], with one study [27] not reporting differences between interventions. Regarding V/P at VT_2_/LT_2_, eight studies did not reveal differences between interventions [25, 28, 29, 32, 52, 55–57], two studies favored POL [8, 26], and three studies [30, 32, 54] suggested superiority of other TIDs.

### Risk of Bias in Studies

The RoB-2 quality assessment showed that all studies were rated as having some concerns due to issues in the randomization process (D1) and selection of the reported results, except for two studies [53, 56] that were rated as having low risk of bias in D1.

**Fig. 2:**
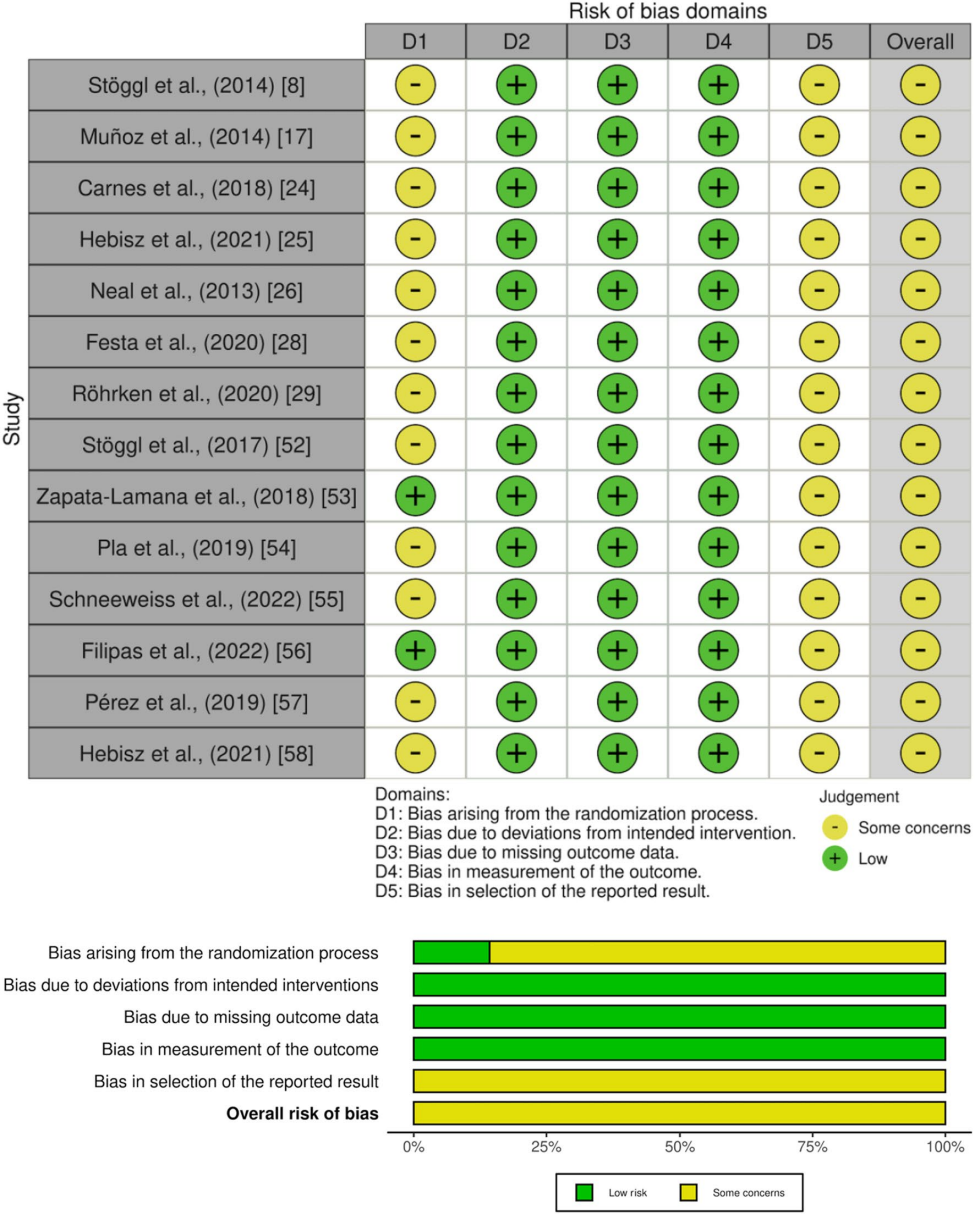
Assessment of risk of bias of randomized trials with RoB-2

**Fig. 3:**
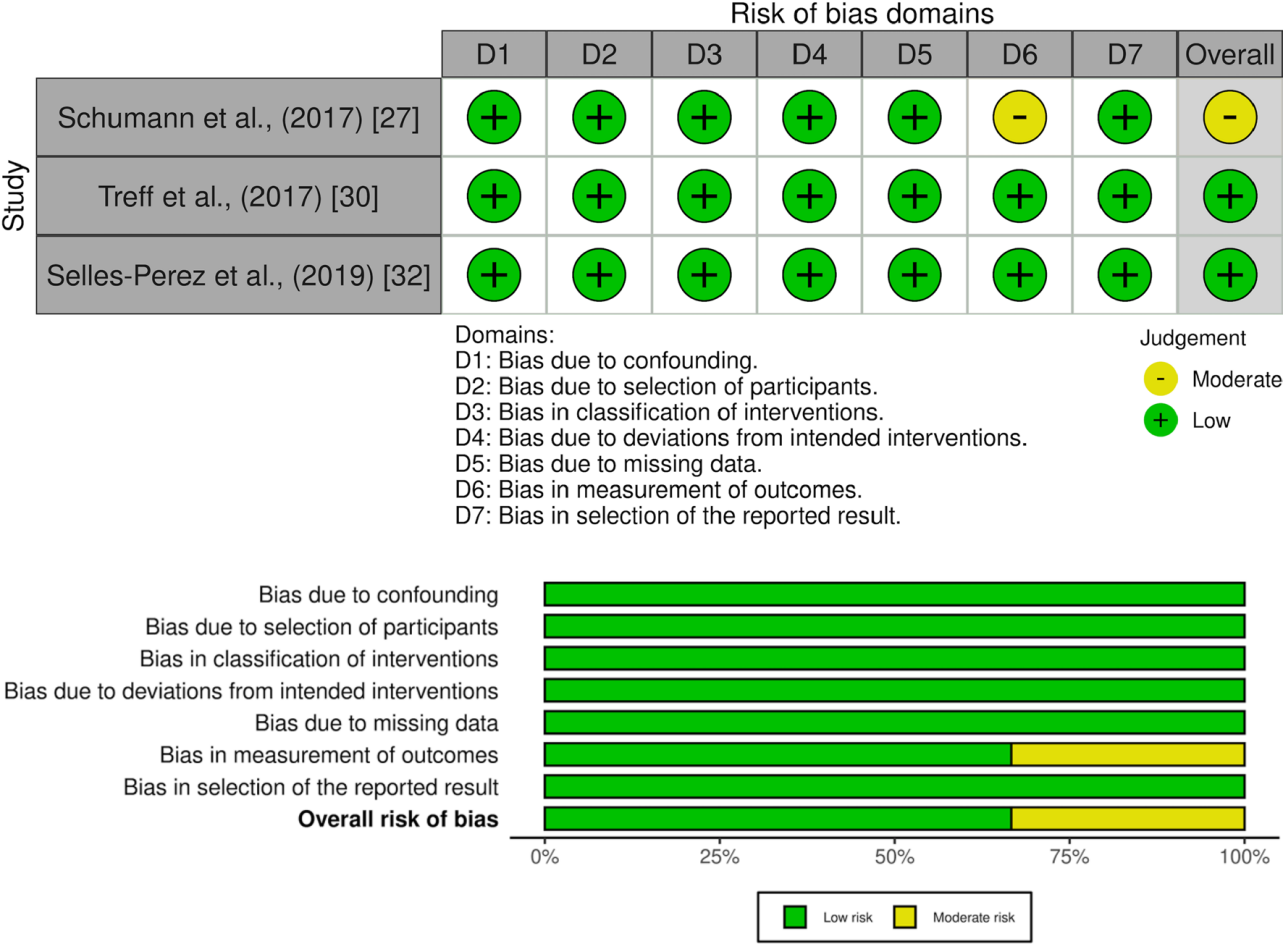
Assessment of risk of bias of non-randomized trials with ROBINS-I

The ROBINS-I quality assessment showed that two studies [11, 32] were rated as having low risk of bias due to having no concerns regarding confounding, selection of participants, classification of interventions, deviations from intended interventions, missing data, measurement of outcomes and selection of the reported results. One study [27] was rated as having some concerns due to moderate bias in the measurement of outcomes. The RoB-2 assessment of all randomized controlled trials and the ROBINS-I assessment of all non-randomized controlled trials are shown in Figs. 2 and 3, respectively.

### Meta-analysis

#### Effect of POL Compared to Other TIDs on* V*O_2_peak

**Fig. 4:**
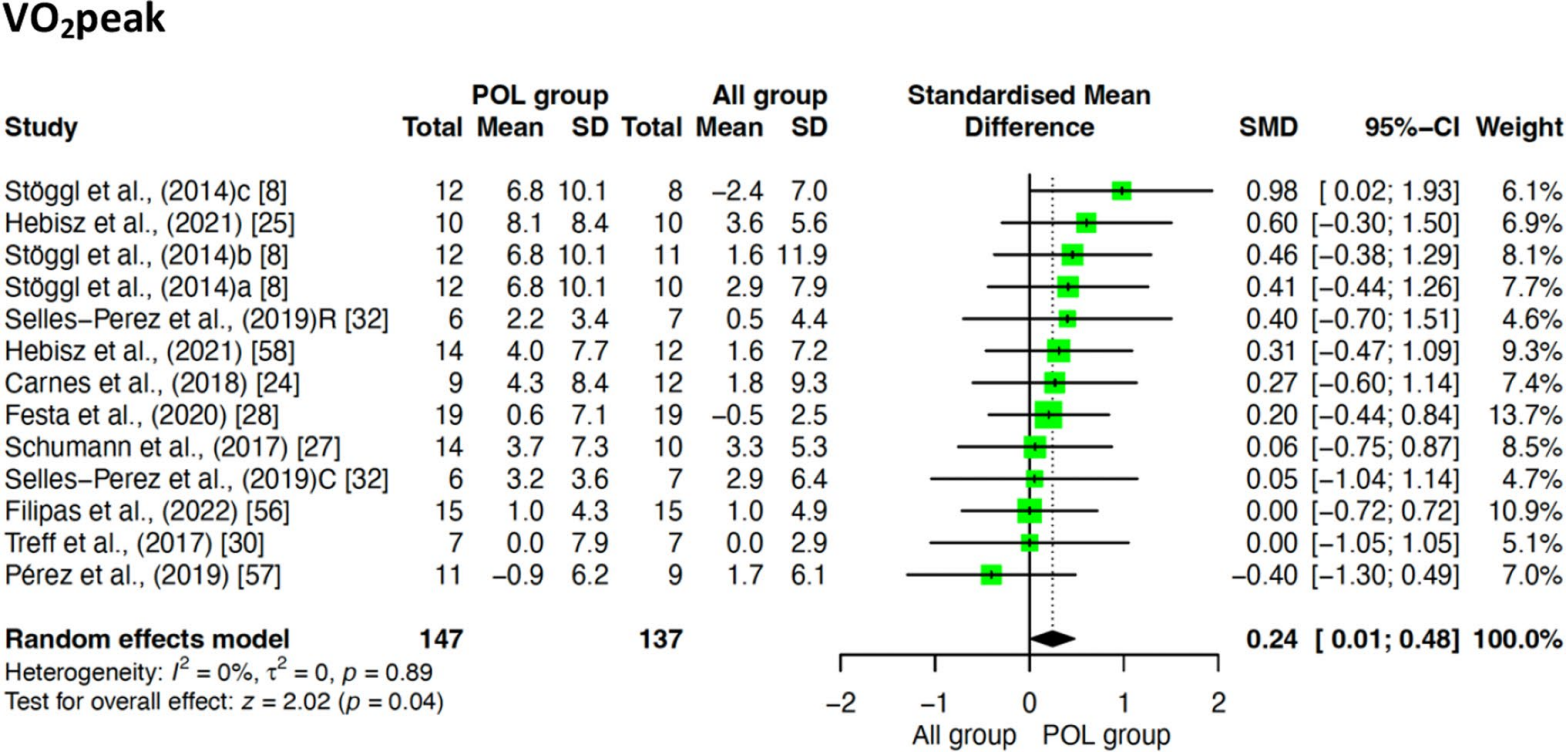
Effect of POL compared to other TIDs on* V*O_2_peak

Figure 4 shows the pooled effect estimates of POL compared to other TIDs on * V*O_2_peak. POL was shown to be superior to other TIDs in the improvement of * V*O_2_peak, although with a small effect size (SMD = 0.24; 95% confidence interval (CI) 0.01, 0.48; *z* = 2.02; *p* = 0.040).

#### Effect of POL Compared to Other TIDs on TT, TTE, and V/P at VT_2_/LT_2_

**Fig. 5:**
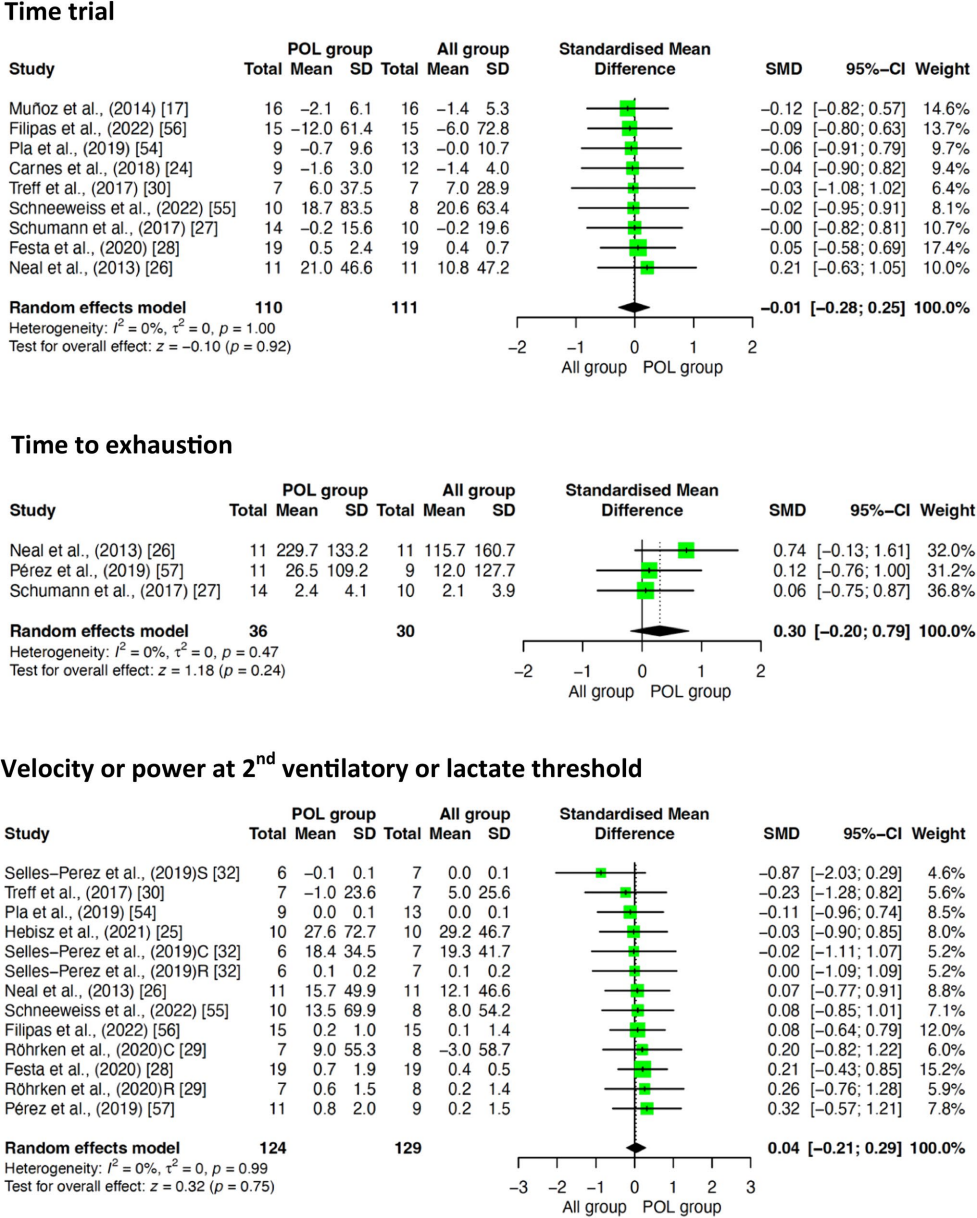
Effect of POL compared to other TIDs on TT, TTE, and V/P at VT_2_/LT_2_

POL in comparison to other TIDs was not shown to induce significant improvements on any of the study secondary outcomes, namely TT (SMD = – 0.01; 95% CI -0.28, 0.25; z =  − 0.10; *p* = 0.92), TTE (SMD = 0.30; 95% CI – 0.20, 0.79; z = 1.18; *p* = 0.24), and V/P at VT_2_/LT_2_ (SMD = 0.04; 95% CI – 0.21, 0.29; *z* = 0.32; *p* = 0.75); see Fig. 5.

#### Comparison of the Effect of POL Versus THR, PYR, HIIT, and CG

No significant differences were identified when comparisons between POL and each of the other TIDs were performed for any of the primary or secondary outcomes of the study. Detailed results are displayed on Table 2.

**Table 2:** Sub analysis of the effect of POL vs. THR, POL vs. PYR, POL vs. HIIT, POL vs. CG

Intervention groups	*V*O_2_max/peak				TT				TTE				V/P at VT_2_/LT_2_			
	*N*	SMD (95% CI)	*I*^2^	*Z* (*p*)	*N*	SMD (95% CI)	*I*^2^	*Z* (*p*)	*N*	SMD (95% CI)	*I*^2^	*Z* (*p*)	*N*	SMD (95% CI)	*I*^2^	*Z* (*p*)
POL vs. THR	78	0.24 (– 0.46; 0.94)	53%	0.67 (0.51)	114	0.01 (– 0.36; 0.38)	0%	0.07 (0.94)	42	0.43 (– 0.18; 1.05)	0%	1.38 (0.17)	132	0.15 (– 0.19; 0.50)	0%	0.88 (0.38)
POL vs. PYR	70	0.08 (– 0.39; 0.55)	0%	0.34 (0.73)	44	– 0.07 (– 0.66; 0.52)	0%	– 0.23 (0.82)	–	–	–	–	83	– 0.14 (– 0.57; 0.30)	0%	– 0.61 (0.54)
POL vs. HIIT	43	0.34 (– 0.27; 0.95)	0%	1.10 (0.27)	–	–	–	–	–	–	–	–	–	–	–	–
POL vs. CG	93	0.34 (– 0.07; 0.76)	0%	1.63 (0.10)	42	– 0.01 (– 0.62; 0.60)	0%	– 0.04 (0.97)	–	–	–	–	38	0.03 (– 0.61; 0.66)	0%	0.08 (0.94)

*CG* control group, *HIIT* high-intensity interval training, *P*O*L* polarized training, *PYR* pyramidal Training, *THR* threshold training, *TT* time trial, *TTE* time to exhaustion, V*/P at VT*_2_*/LT*_2_ velocity or power at second ventilatory or lactate threshold, *V*O_2_max*/peak* maximal/peak oxygen uptake, *SMD* standardized mean difference, *I*_2_* (p)* heterogeneity and p-value, *Z (p)* test for overall effect and p-value

#### Effect of POL Compared to Other TIDs According to Intervention Duration (< 12 and ≥ 12 Weeks)

**Fig. 6:**
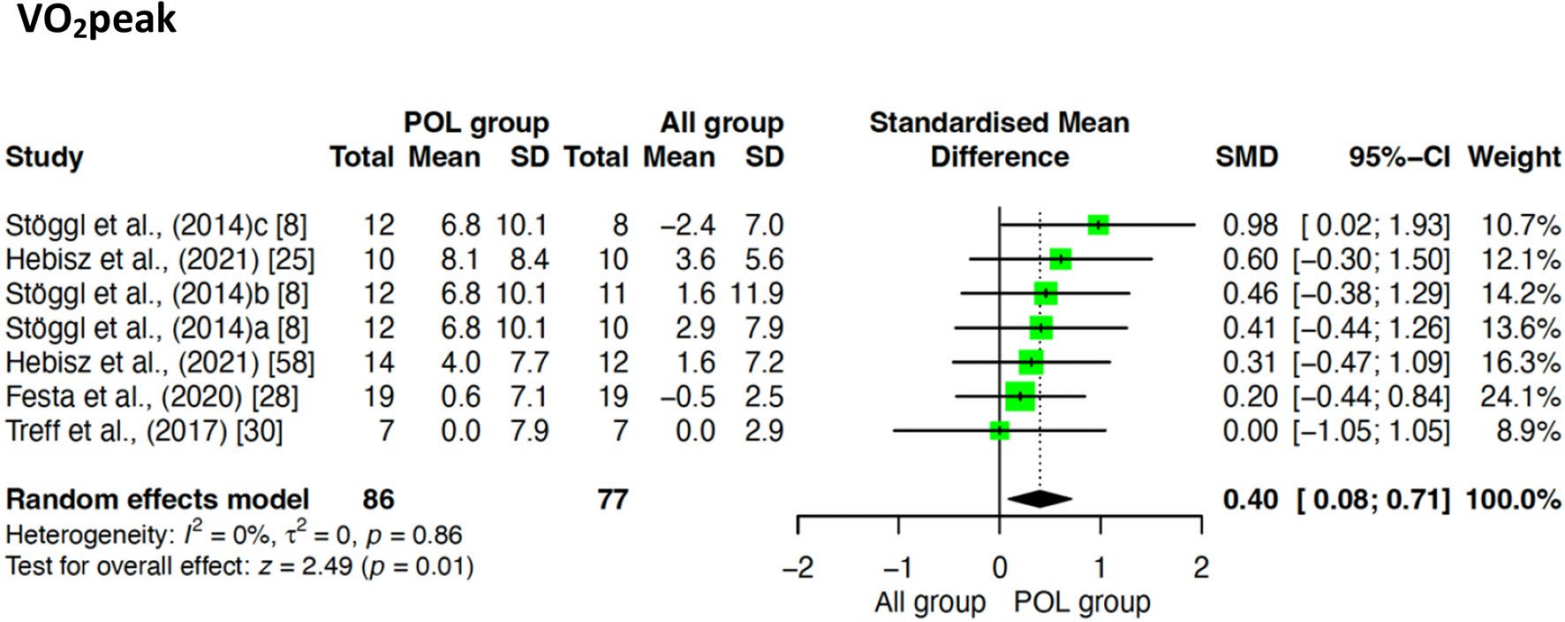
Sub-analysis of POL vs ALL < 12 weeks

A sub‐analysis according to training intervention duration was also performed and is shown in Table 3, in which interventions were divided into two categories: < 12 weeks’ duration and ≥ 12 weeks’ duration. POL was only shown to be superior to other TIDs on* V*O_2_peak when intervention duration was < 12 weeks (SMD = 0.40; 95% CI 0.08, 0.71; *z* = 2.49; *p* = 0.01; Fig. 6). For TT (*p* = 0.98), and V/P at VT_2_/LT_2_ (*p* = 0.65) there were no differences between POL and other TIDs according to intervention duration. When training duration was ≥ 12 weeks, POL was not shown to be superior for any of the study outcomes (VO_2_peak, *p* = 0.83; TT, *p* = 0.84; TTE, *p* = 0.78; V/P at VT_2/_LT_2_, *p* = 0.93).

**Table 3:** Sub analysis of the effect of POL compared to other TIDs according to intervention duration and to athlete level

Intervention groups	*V*O_2_max/peak				Time trial				Time to exhaustion				V/P at VT_2/_LT_2_			
	*N*	SMD (95% CI)	*I*^2^	*Z* (*p*)	*N*	SMD (95% CI)	*I*^2^	*Z* (*p*)	*N*	SMD (95% CI)	*I*^2^	*Z* (*p*)	*N*	SMD (95% CI)	*I*^2^	*Z* (*p*)
POL vs. ALL (< 12 weeks)	163	0.40 (0.08; 0.71)	0%	2.49 (0.01)*	146	0.00 (– 0.32; 0.33)	0%	0.03 (0.98)	–	–	–	–	164	0.07 (– 0.24; 0.38)	0%	0.46 (0.65)
POL vs. ALL (≥ 12 weeks)	121	0.04 (– 0.32; 0.40)	0%	0.22 (0.83)	87	– 0.14 (– 0.56; 0.29)	0%	– 0.63 (0.53)	44	0.09 (– 0.51; 0.68)	0%	0.28 (0.78)	89	– 0.02 (– 0.44; 0.40)	0%	– 0.09 (0.93)
POL vs. ALL (Highly Trained)	118	0.46 (0.10; 0.82)	0%	2.51 (0.01)*	54	– 0.04 (– 0.58; 0.50)	0%	– 0.14 (0.89)	–	–	–	–	74	– 0.06 (– 0.52; 0.40)	0%	– 0.27 (0.79)
POL vs. ALL (Trained)	159	0.08 (– 0.23; 0.39)	0%	0.50 (0.62)	167	– 0.00 (– 0.31; 0.30)	0%	– 0.03 (0.98)	66	0.30 (– 0.20; 0.79)	0%	1.18 (0.24)	179	0.08 (– 0.21; 0.38)	0%	0.55 (0.58)
POL vs. ALL (TT < 12 min)	129	–	–	–	116	– 0.00 (– 0.37; 0.37)	0%	– 0.01 (1.00)	–	–	–	–	–	–	–	–
POL vs. ALL (TT ≥ 12 min)	159	–	–	–	105	– 0.03 (– 0.41; 0.36)	0%	– 0.13 (0.89)	–	–	–	–	–	–	–	–

*ALL* other training intensity distribution, *P*O*L* polarized training, *TT* time trial, *TTE* time to exhaustion, *V/P at VT*_2_*/LT*_2_ velocity or power at second ventilatory or lactate threshold, *V*O_2_max*/peak* maximal/peak oxygen uptake, *SMD* standardized mean difference, *I*_2_* (p)* heterogeneity and p-value, *Z (p)* test for overall effect and p-value

^*^Statistical significance: *p* < 0.05

#### Effects of POL Compared to Other TIDs According to Athlete Level

**Fig. 7:**
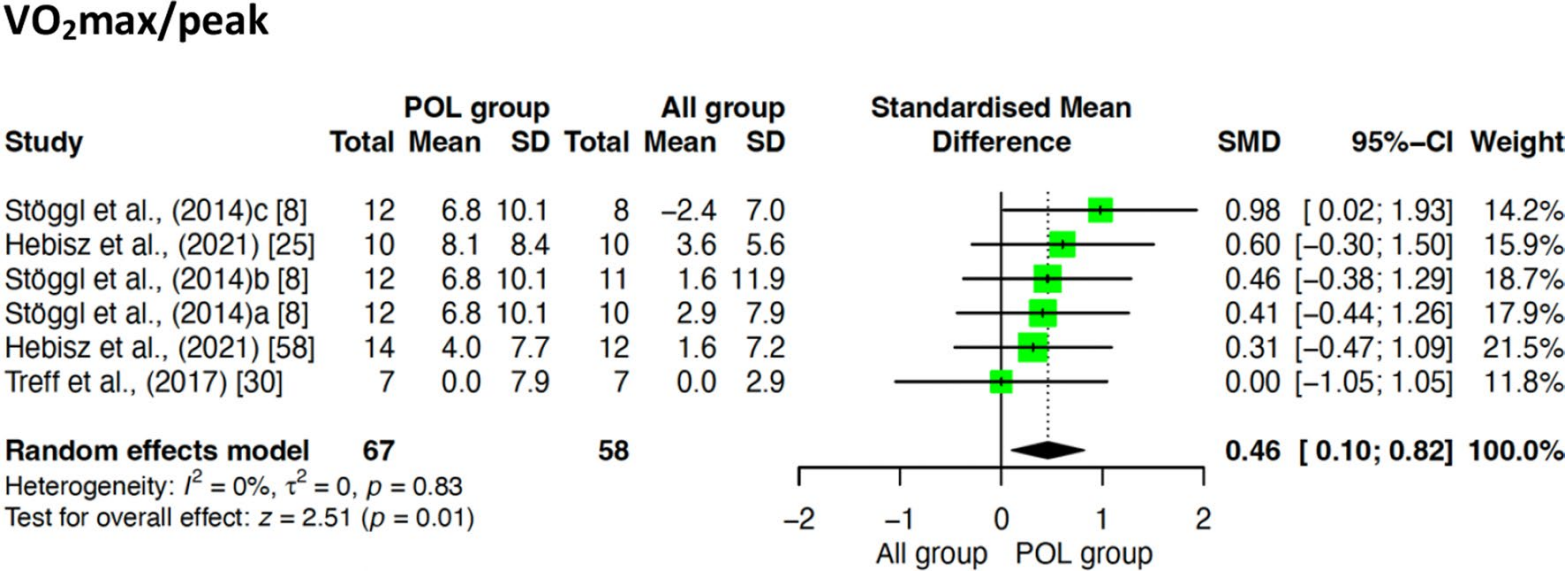
Sub-analysis of POL vs. ALL according to competitive level—highly trained/national level athletes

A sub-analysis comparing the effect of endurance performance starting level (highly trained/national level *vs* trained/developmental) [44] on the selected outcomes was performed (Table 3). In highly trained/national level athletes, POL was superior to other TIDs on the improvement of *V*O_2_peak (SMD = 0.46; 95% CI 0.10, 0.82; *z* = 2.51; *p* = 0.01), but not for TT (*p* = 0.89), and V/P at VT_2_/LT_2_ (*p* = 0.79). For trained/developmental athletes, there were no differences between POL and other TIDs for any of the outcomes assessed (VO_2_peak: *p* = 0.82; TT: *p* = 0.98; TTE: *p* = 0.24; V/P at VT_2_/LT_2_: *p* = 0.58; Fig. 7).

#### Effects of POL Compared to Other TIDs According to TT Duration (< 12 min vs. ≥ 12 min)

A sub‐analysis according to TT duration was also performed and is shown in Table 3, in which interventions were divided into two categories, < 12 min versus ≥ 12 min duration. There were no differences in TT performance between POL and other TIDS, irrespective of TT test duration (< 12 min: *p* = 1.00; > 12 min: *p* = 0.89).

#### Effect of Sex

Although in the pre-registration of this work a sub-analysis comparing the effect of POL versus other TIDs between males and females was proposed (sex effect on the adaptation to POL), it was not possible to perform this analysis due to insufficient sex reporting in the included studies.

### Sensitivity Analysis

All the analyses performed revealed low or inexistence of heterogeneity. The analysis with the highest heterogeneity was a secondary analysis of POL versus THR for *V*O_2_peak (*I*^2^ = 53%). A sensitivity analysis for this comparison was performed by removing one study at a time. The study that was shown to contribute the most to heterogeneity was the one by Stöggl et al. [8]. Nevertheless, removal of this study did not affect the results (*p* = 0.95) (see Appendix S3 of the ESM).

### Certainty of the Evidence

According to the GRADE approach, all outcomes were classified as having a high certainty of evidence (Appendix S4 of the ESM).

### Publication Bias

Publication bias was assessed for studies comparing athletes performing POL vs other TIDs on* V*O_2_peak and V/P at VT_2_/LT_2_. A non-significant publication bias was revealed by both the funnel plot symmetry and the Egger's test result adjusted for *V*O_2_peak (bias coefficient = – 0.042 (intercept); *p* = 0.632), and V/P at VT_2_/LT_2_ (bias coefficient = – 0.708 (intercept); *p* = 0.092). Detailed results are depicted in Appendix S5 of the ESM.

## Discussion

This study aimed to systematically review and meta-analyze the evidence comparing the effect of POL with other TIDs on endurance performance. POL was found to be superior to other TIDs for* V*O_2_peak improvement, although with a small magnitude of effect (SMD = 0.24 [95% CI 0.01, 0.48]; *z* = 2.02 (*p* = 0.040); *n* = 284; *I*^2^ = 0%). Regarding the secondary outcomes, there was no evidence of superiority of POL compared to other TIDs.

To date, only one systematic review with meta-analysis [33] have compared POL with THR, but this included only TT as a surrogate of endurance performance. In addition, this meta-analysis contained only three studies due to the scarcity of data available at that time. Their results suggested superiority of POL compared to THR for TT improvement, which is in opposition to our findings. This disparity is due to the difference in the number of studies included in the analysis. The increased interest in POL has led to a surge in the number of experimental studies investigating this TID. Consequently, our study included a sample of *n* = 437 participants compared to *n* = 112 in the Rosenblat et al. study [33]. Another important difference was the inclusion of other TIDs as well as a set of variables strongly correlated with endurance performance such as *V*O_2_peak, TT, TTE and V/P at VT_2_/LT_2_, other than TT, which allows for a much more thorough understanding of the effectiveness of POL.

Our results showed that *V*O_2_peak was higher with POL compared to other TIDs. The results displayed a low heterogeneity but a small effect size. POL may have been more effective in promoting *V*O_2_peak adaptations as it exposes athletes to a combination of low- and high-intensity exercise, which appears to be particularly suited to the development of central and peripheral aerobic adaptations [23]. The main central adaptation to endurance training is the increase in cardiac output, which results mainly from increases in blood volume, left ventricle end-diastolic volume, and myocardial contractility [59, 60]. Of these adaptations, the one that most likely contributes to improvements in *V*O_2_peak in studies with a short duration, such as those included in our systematic review, is an increase in blood volume. It has been shown that low-intensity aerobic training is effective in increasing plasma volume and cardiac output [61]. However, high-intensity training appears to be an even more effective strategy for this purpose [62, 63]. Previous studies have shown that intensity is a crucial variable in exercise-induced hypervolemia, and that higher exercise intensities seem particularly effective at inducing rapid elevations in plasma volume [64, 65]. For instance, adding a short period of high intensity exercise, between 90 and 95% *V*O_2_peak, to well-trained runners, significantly increased their blood volume by 4% [66]. Increases in blood volume of 10% 24 h after a single exercise session at 85% VO_2_peak has also been reported [67]. Evidence suggesting that high-intensity exercise rapidly increases blood volume is also in accordance with the findings of our sub-analysis showing that it was with shorter interventions (< 12 weeks) that POL was particularly effective compared to other TIDs for improving *V*O_2_peak (SMD = 0.40; 95% CI 0.08, 0.71; z = 2.49; *p* = 0.01).

Improvements in *V*O_2_peak also depend, to a large extent, on peripheral skeletal muscle adaptations favoring capillary oxygen extraction and use by fiber mitochondria [68]. Although it is well demonstrated [69] that high-volume, low-intensity training favors mitochondrial biogenesis [70], increases lactate oxidation rate [71] and type I muscle fibers capillarization [68, 72], there is also evidence that high intensity is a key factor in peroxisome proliferator-activated receptor-gamma coactivator-1 alpha (PGC-1α) activation and mitochondrial biogenesis [73]. A single high-intensity exercise bout was shown to induce greater elevations in PGC-1α mRNA compared to low-intensity exercise [74]. This might be explained by the fact that high-intensity exercise is a major stimulus for ATP depletion and accumulation of ADP and AMP, which will thereby activate PGC1-α and trigger mitochondrial biogenesis [75–77]. Training at higher intensities has also been shown to lead to faster improvements in endurance performance [78, 79] and to faster peripheral adaptations compared to training at moderate or low intensity [80]. This may also be due to the greater recruitment of type II muscle fibers, which positively affects their oxidative capacity [81–83]. For instance, high-intensity exercise seems to induce superior adaptations in type II fibers’ oxidative metabolism compared to low-intensity aerobic training [84]. In addition, when high-intensity exercise is performed at an intensity greater than *V*O_2_peak, the oxidative capacity of type IIx muscle fibers is enhanced [85]. Collectively, this evidence suggests that adding high-intensity exercise to the training regimen favors a faster development of both central and peripheral adaptations, thereby optimally enhancing *V*O_2_peak. This is in agreement with our sub-analysis showing that POL training is particularly effective in inducing faster increases at *V*O_2_peak. Nevertheless, the advantage of POL seems to wane for interventions longer than 12 weeks, suggesting that subjects who undergo other TIDs might also develop adaptations to the same extent as those induced by POL, but more slowly. For this reason, POL might be a more interesting strategy to induce faster improvements in *V*O_2_peak, such as for instance in prehabilitation exercise contexts [86].

Despite high *V*O_2_peak being one of the determinants of endurance performance [87], other variables are also important in this context [61, 88]. Anaerobic threshold, which translates into the ability to maintain high workloads without exponentially increasing blood lactate concentration [35], is a variable that is more strongly correlated with endurance sports performance [89]. Interestingly, our results suggest that, for this variable, there is no evidence of superiority of POL compared to other TIDs (*p* = 0.75).

Exercise intensity at VT_1_ and, especially, at VT_2_ are major determinants of endurance performance since VT_2_ marks the intensity above which lactate concentration consistently rises, hindering the ability to tolerate the exercise intensity for a long period. Endurance training plays an important role in reducing blood lactate concentration for a given exercise intensity [60]. This reduction seems to be a consequence of a lower rate of muscle glycogen utilization [90], accelerated O_2_ consumption kinetics [91], and the ability to effectively remove blood lactate [92]. The most plausible physiological rationale for increasing velocity at the anaerobic threshold is increased lactate clearance [93]. After both low-intensity [94] and high-intensity [95] endurance training interventions, concentrations of monocarboxylic transporters (MCT), namely MCT1 and MCT4 seem to increase. A high abundance of MCT facilitates transport of lactate and hydrogen ions and increases muscle lactate clearance [96, 97]. In fact, this is verifiable by analyzing the expression of MCT1 in well-trained subjects, which is much higher compared to less trained subjects [98]. Furthermore, the rate of lactate removal after maximal exertion correlates with MCT1 expression, thereby favoring high ATP utilization rates without major increases in blood lactate [99]. Considering that both low-intensity and high-intensity exercise seem to induce similar adaptations in the mechanisms involved in lactate production and removal, it is not surprising that several studies using different exercise training intensities, such as threshold training [26, 100], training above the anaerobic threshold [101, 102], and POL [26, 103], were all effective in improving the anaerobic threshold, which is in agreement with our findings that POL is similar to other TIDs regarding V/P at VT_2_/LT_2_.

TT is a variable highly correlated with endurance performance [104]. In our study, there was no evidence of superiority of POL compared to other TIDs in the improvement of TT (*p* = 0.92). Nevertheless, the studies included in our meta-analysis displayed a high variability in terms of TT distances, ranging from 100 m [54] to 40 km [26], which correlate very differently with performance in aerobic activities, and therefore some of these results might not necessarily reflect adaptations of the aerobic metabolism. TT in endurance activities is highly dependent on *V*O_2_peak and, especially for longer distances, on LT and running economy [87, 105–107]. Considering our findings that POL is similar to other TIDs regarding improvements in V/P at VT_2_/LT_2_ and that POL is only able to marginally improve VO_2_peak, especially for shorter duration interventions, it is not surprising that the results from our meta-analysis suggest that TT can be similarly improved by several TIDs even irrespective of TT duration.

Our meta-analysis included only three studies [26, 27, 57] analyzing TTE. The protocols by Perez et al. [57] and Schumann et al. [27] consisted of incremental tests until exhaustion, while Neal et al. [26] performed a protocol at 95% of peak power output in cycle ergometer to exhaustion. In these types of tests, in which athletes perform exercise at high intensity, there is a marked production and accumulation of lactate and hydrogen ions [108]. Consequently, muscle pH will drop dramatically [109] and, therefore, effective pH regulation, which is highly dependent on skeletal muscle buffering capacity [110], is a crucial factor for prolonging TTE. Interestingly, since high-intensity exercise recruits a substantial portion of fast-twitch muscle fibers, this has been shown to favor resistance to fatigue at higher intensities, therefore prolonging TTE [82, 111]. However, in recreational runners, continuous aerobic training at intensities between 60 and 80% *V*O_2_peak has also been shown to effectively improve TTE by increasing cardiac output and oxidative enzyme activity [112]. Therefore, different physiological adaptations, induced by different exercise intensities, seem to be able to effectively improve TTE in endurance activities, explaining the absence of differences between POL and other TIDs regarding improvements in TTE identified in our meta-analysis.

Knowing a priori that the starting endurance performance level of the subjects could be a differentiating factor in the magnitude of the response to the training stimulus, we carried out a sub-analysis of our variables of interest according to the initial endurance performance level of the subjects (highly trained/national level vs*.* trained/developmental). Our results showed that baseline performance level significantly influenced the effectiveness of endurance training type with only highly trained/national level athletes showing higher improvements in VO_2_peak in response to POL compared to other TIDs. This finding is in agreement with previous studies showing that adding a period of high-intensity exercise to even well-trained athletes effectively induces several hematological adaptations [66] that could favor *V*O_2_peak increases [113]. Nevertheless, studies including highly trained athletes were also those that had a shorter duration (< 12 weeks). Therefore, it is not possible to disentangle whether the observed effect was due to training level or to the concomitant effect of intervention duration. Future studies should be performed to specifically address this question.

One of the main limitations of this study is that several of the included reports did not disclose the percentage of TID. Although the authors classified the training model as POL or PYR, without data on the %TID performed at each zone, it is problematic to robustly state which model was in fact followed. Another necessary criticism is the under-reporting of weekly TRIMPs or variables such as volume, intensity, and frequency of the entire training program. Therefore, it is possible that groups might have differed not just in intensity but also in other crucial variables that were not accounted for. In addition, several studies lacked adequate description of the training program variables, namely weekly frequency, type of sessions, and robust measures of volume and intensity. It is also noteworthy to mention that although our study included several important variables for endurance performance, the success in endurance activities also depends on other aspects that were not assessed. For instance, Seiler et al. [114] and Boullosa et al. [115, 116] argue that the characteristics of POL favor a reduction in fatigue, and therefore that when training volumes are substantially high, POL may be a superior strategy for reducing the risk of overtraining. Consequently, although our study identified only marginal benefits of POL in improving variables related to endurance performance, future studies should further investigate other determinants of endurance performance success in a more ecological context, namely those related to recovery [117–120].

## Conclusions

In conclusion, the results of our systematic review and meta-analysis suggest, with high certainty of evidence, that POL is superior to other TIDs for the improvement of *V*O_2_peak, but with a small effect size, and particularly for shorter duration interventions and in the case of highly trained/national level athletes. There was, however, no evidence of superiority of POL regarding TT, TTE and V/P at VT_2_/LT_2_. POL could be a more effective strategy to increase *V*O_2_peak in a short period of time, particularly in highly trained athletes.

These results should raise exercise physiologists’ and coaches’ attention to the importance of including POL TID regiments in pre-competitive phases, particularly in endurance sports that are highly dependent on aerobic power, since our results suggest that a reduced number of weeks under this TID could lead to faster improvements in *V*O_2_peak.

### Supplementary Information

Below is the link to the electronic supplementary material.

Supplementary file1 (DOCX 33 KB)

Supplementary file2 (DOCX 5979 KB)

Supplementary file3 (DOCX 2946 KB)

Supplementary file4 (DOCX 22 KB)

Supplementary file5 (DOCX 1012 KB)

## Declarations

### Data Availability

Data could be made available for purposes deemed to be appropriate upon contact with the corresponding author.
