# Supplementary material for: Comparison of Polarized Versus Other Types of Endurance Training Intensity Distribution on Athletes’ Endurance Performance: A Systematic Review with Meta-analysis
Source: Sports Med. 2024 May 8;54(8):2071–95. doi: 10.1007/s40279-024-02034-z (PMC11329428; doi:10.1007/s40279-024-02034-z)
Supplement: Supplementary file 2 — Supplementary file2 (DOCX 5979 KB) [file 40279_2024_2034_MOESM2_ESM.docx]

**Online Resource S2**

**Title: Comparison of Polarized *vs* Other Types of Endurance Training Intensity Distribution on Athletes Endurance Performance: A Systematic Review with Meta-Analysis**

**Journal:** Sports Medicine.

**Authors:** Pedro Oliveira^1,2^, Giorjines Boppre^1,2,3^, Hélder Fonseca^1,2^

^1^ Research Centre in Physical Activity, Health and Leisure (CIAFEL), Faculty of Sport, University of Porto, Portugal

^2^ Laboratory for Integrative and Translational Research in Population Health (ITR), Porto, Portugal

^3^ Human Motricity Research Center, University Adventista, Chillan, Chile

**Corresponding author:** Pedro Oliveira ([up201807240@fade.up.pt](mailto:up201807240@fade.up.pt))

**Electronic Supplementary Material Appendix, S2.** Preprints of search strategy


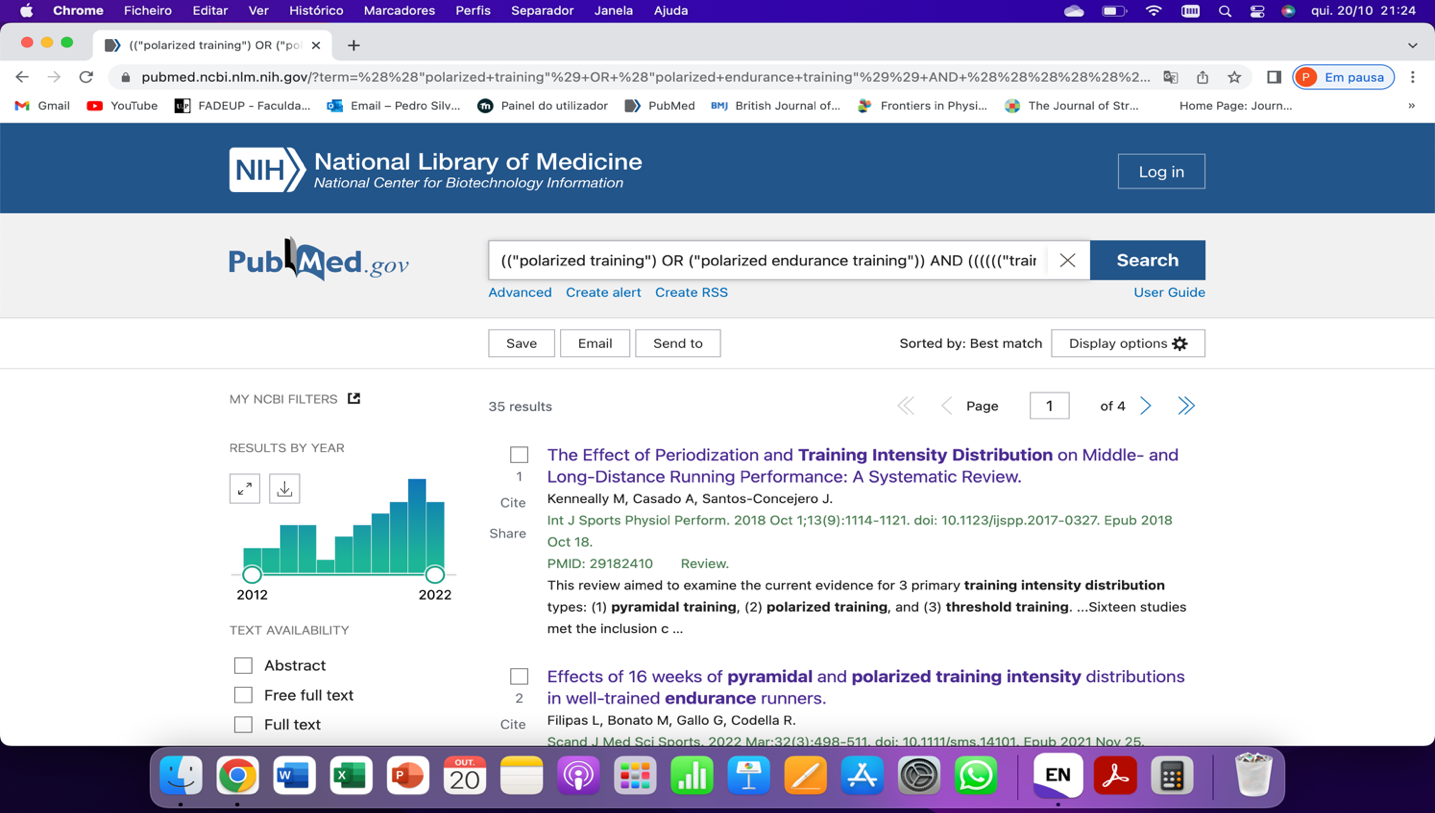

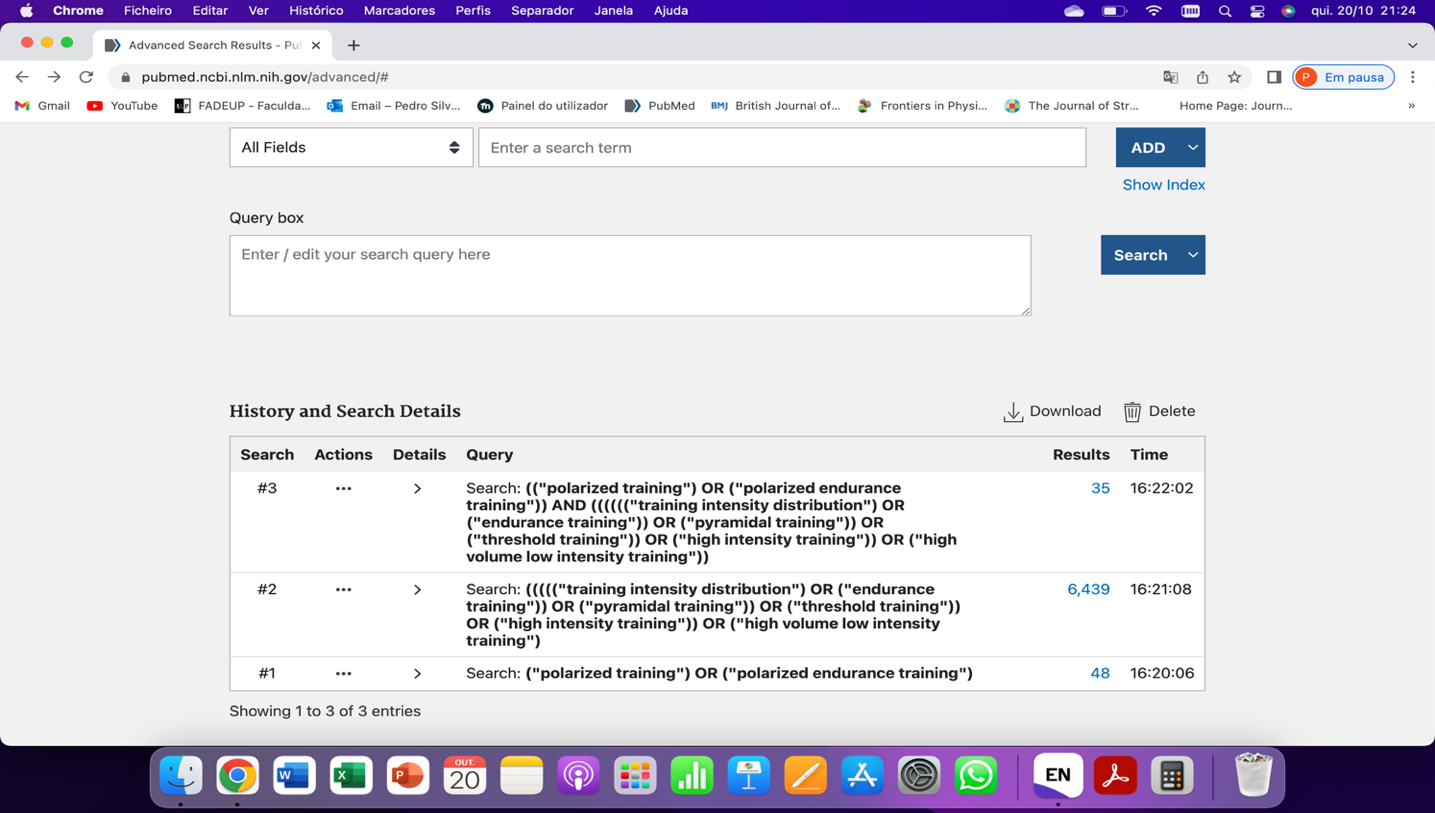


**Fig. S1** Preprint search strategy PubMed


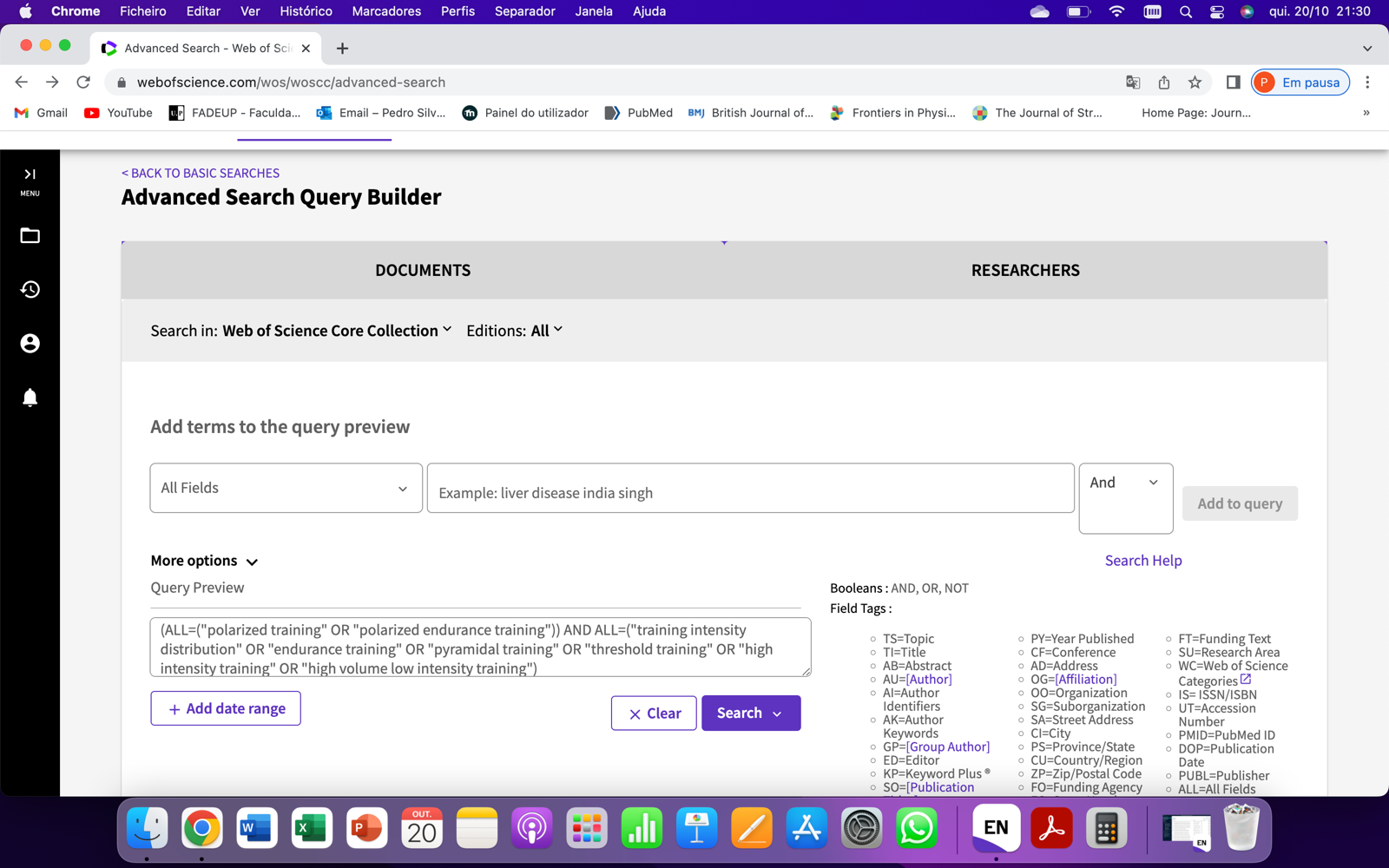

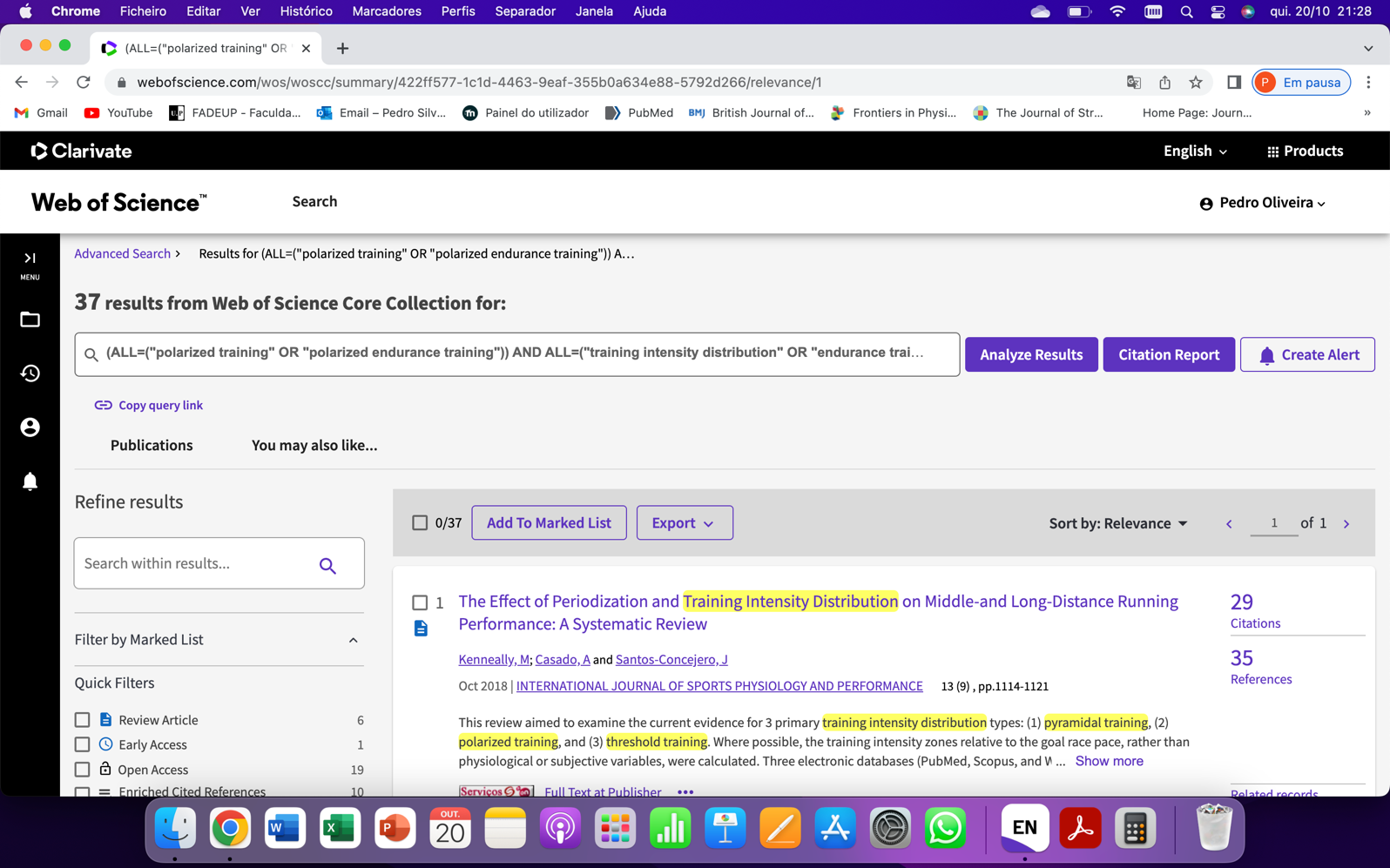


**Fig. S2** Preprint search strategy Web of Science

**Fig. S3** Preprint search strategy Scopus

**
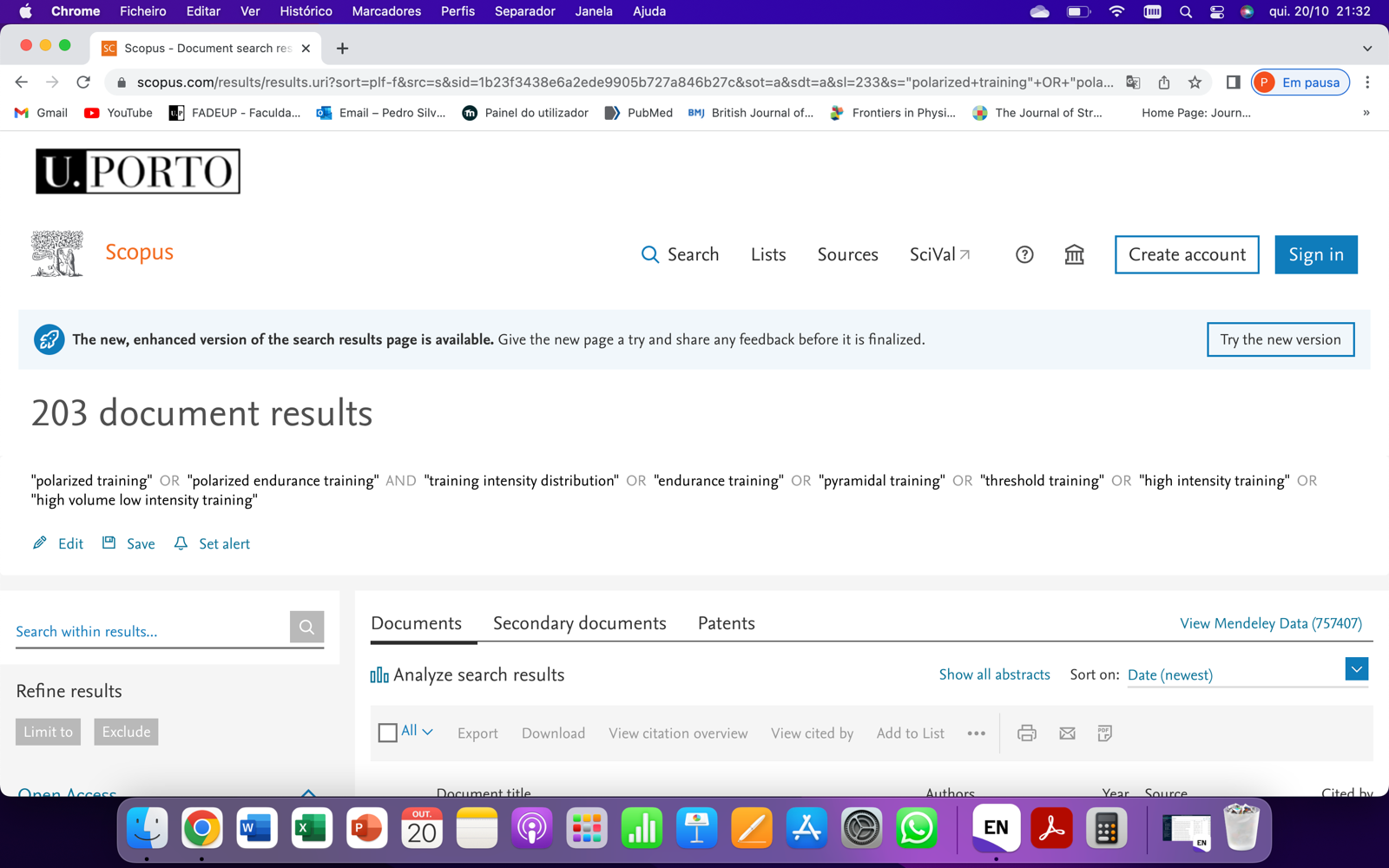
**
