# Supplementary material for: Comparison of Polarized Versus Other Types of Endurance Training Intensity Distribution on Athletes’ Endurance Performance: A Systematic Review with Meta-analysis
Source: Sports Med. 2024 May 8;54(8):2071–95. doi: 10.1007/s40279-024-02034-z (PMC11329428; doi:10.1007/s40279-024-02034-z)
Supplement: Supplementary file 3 — Supplementary file3 (DOCX 2946 KB) [file 40279_2024_2034_MOESM3_ESM.docx]

**Online Resource 3**

**Title: Comparison of Polarized *vs* Other Types of Endurance Training Intensity Distribution on Athletes Endurance Performance: A Systematic Review with Meta-Analysis**

**Journal:** Sports Medicine.

**Authors:** Pedro Oliveira^1,2^, Giorjines Boppre^1,2,3^, Hélder Fonseca^1,2^

^1^ Research Centre in Physical Activity, Health and Leisure (CIAFEL), Faculty of Sport, University of Porto, Portugal

^2^ Laboratory for Integrative and Translational Research in Population Health (ITR), Porto, Portugal

^3^ Human Motricity Research Center, University Adventista, Chillan, Chile

**Corresponding author:** Pedro Oliveira ([up201807240@fade.up.pt](mailto:up201807240@fade.up.pt))

**Electronic Supplementary Material Appendix S3.** Sensitivity analysis

a*

**b***

**c***


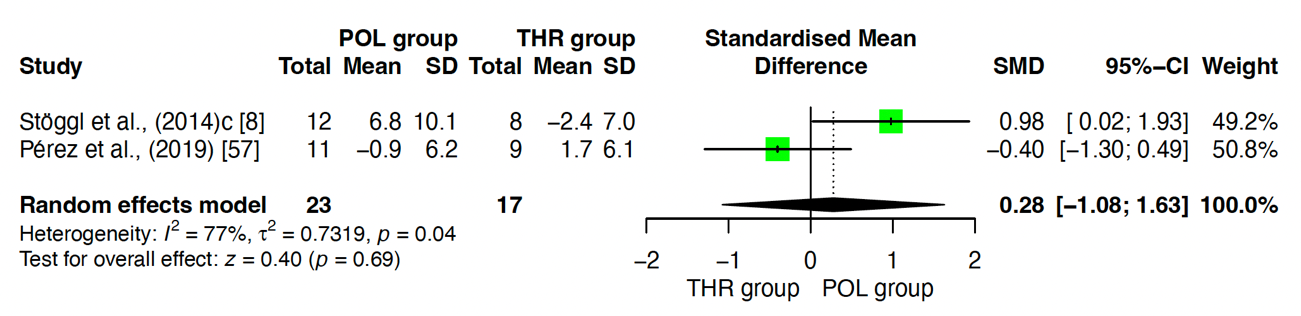

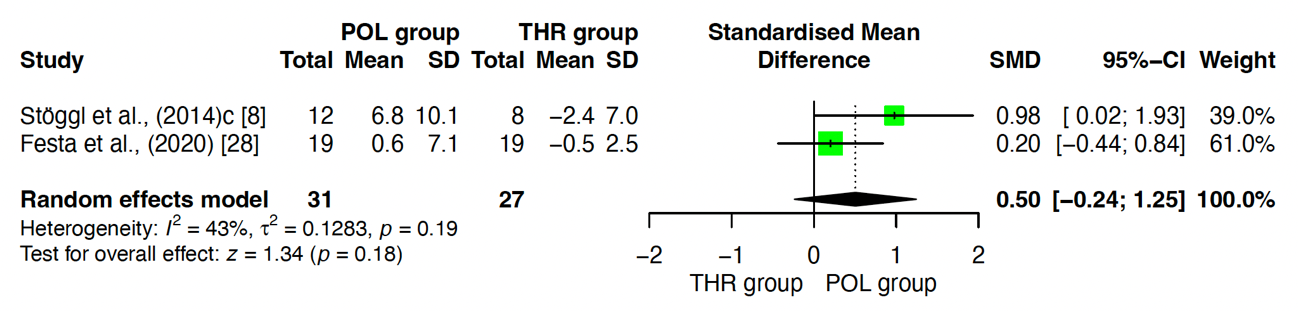

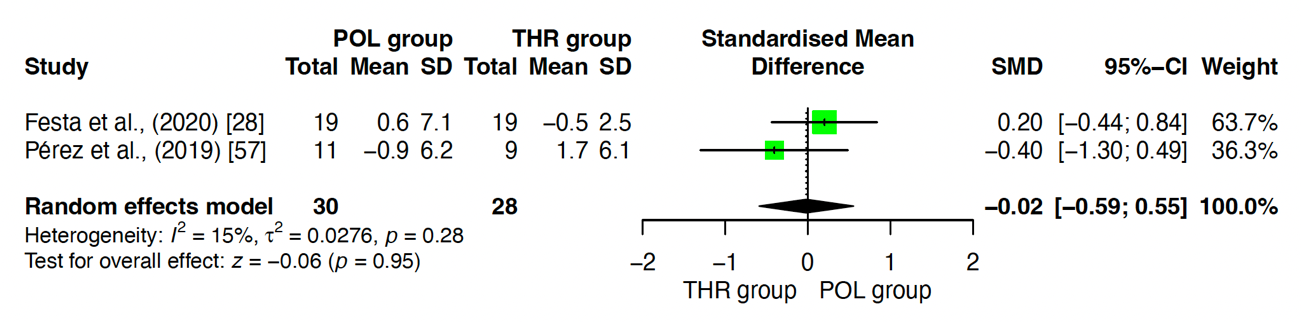


**Fig. S1** Sensitivity analysis of POL vs THR regarding VO_2_peak

Note: A sensitivity analysis for this comparison was performed by removing one study at a time. The study that was shown to contribute the most to heterogeneity was the one by Stoggl et al., (2014)c [7]. Nevertheless, removal of this study did not affect the results (p= 0.95), c*.
