# Supplementary material for: Comparison of Polarized Versus Other Types of Endurance Training Intensity Distribution on Athletes’ Endurance Performance: A Systematic Review with Meta-analysis
Source: Sports Med. 2024 May 8;54(8):2071–95. doi: 10.1007/s40279-024-02034-z (PMC11329428; doi:10.1007/s40279-024-02034-z)
Supplement: Supplementary file 4 — Supplementary file4 (DOCX 22 KB) [file 40279_2024_2034_MOESM4_ESM.docx]

**Online Resource S4**

**Title: Comparison of Polarized *vs* Other Types of Endurance Training Intensity Distribution on Athletes Endurance Performance: A Systematic Review with Meta-Analysis**

**Journal:** Sports Medicine.

**Authors:** Pedro Oliveira^1,2^, Giorjines Boppre^1,2,3^, Hélder Fonseca^1,2^

^1^ Research Centre in Physical Activity, Health and Leisure (CIAFEL), Faculty of Sport, University of Porto, Portugal

^2^ Laboratory for Integrative and Translational Research in Population Health (ITR), Porto, Portugal

^3^ Human Motricity Research Center, University Adventista, Chillan, Chile

**Corresponding author:** Pedro Oliveira ([up201807240@fade.up.pt](mailto:up201807240@fade.up.pt))

**Electronic Supplementary Material Appendix S4.** GRADE – Summary of findings

**Table S1** GRADE - Summary of findings

| Certainty assessment | | | | | | | | № of patients | |  | Certainty |
| --- | --- | --- | --- | --- | --- | --- | --- | --- | --- | --- | --- |
| № of studies | **Study design** | **Risk of bias** | **Inconsistency** | **Indirectness** | **Imprecision** | | **Other considerations** | **POL** | **All** | **Absolute effect (95% CI)** |  |
| VO_2_peak | | | | | | | | | | | |
| 13 | RCT and Non-RCT **^a^** | not serious **^g^** | not serious | not serious | | not serious | none | 147 | 137 | SMD **0.24**  (0.01 to 0.48) | ⨁⨁⨁⨁ High |
| TT | | | | | | | | | | | |
| 10 | RCT and Non-RCT **^b^** | not serious **^h^** | not serious | not serious | | not serious | none | 110 | 111 | SMD  -**0.01**  (-0.28 to 0.25) | ⨁⨁⨁⨁ High |
| TTE | | | | | | | | | | | |
| 3 | RCT and Non-RCT **^c^** | not serious **^i^** | not serious | not serious | | not serious | none | 36 | 30 | SMD **0.3**  (-0.2 to 0.79) | ⨁⨁⨁⨁ High |
| V/P at  VT_2/_LT_2_ | | | | | | | | | | | |
| 13 | RCT and Non-RCT **^d^** | not serious **^j^** | not serious | not serious | | not serious | none | 124 | 129 | SMD **0.04**  (-0.21 to 0.29) | ⨁⨁⨁⨁ High |

Abbreviations: All: Other Training Intensity Distributions; CI: confidence interval; POL: Polarized Training; SMD: standardized mean difference; TT: Time trial; TTE: Time to exhaustion; V/P at VT_2_/LT_2_: Velocity or Power at 2^nd^ ventilatory or lactate threshold; VO_2_peak: Peak oxygen uptake.

Notes: a) Nine studies out of 13 are randomized controlled trials (RCTs); b) 8 studies out of 10 are RCTs; c) 2 studies out of 3 are RCTs; d) 9 studies out of 13 are RCTs; g) Of the 4 non-RCT studies, one was rated as moderate and 3 as low risk of bias assessed by ROBINS-I, and 9 studies were rated as some concerns risk of bias assessed by RoB-2; h) Of the 2 non-RCT studies, 1 was rated as moderate and 1 as low risk of bias assessed by ROBINS-I, and 8 studies were rated as some concerns risk of bias assessed by RoB-2; i) One non-RCT study was rated as moderate risk of bias assessed by ROBINS-I, and 2 RCTs studies were rated as some concerns risk of bias assessed by RoB-2; j) Of the 4 non-RCT studies, one was rated as moderate and 3 as low risk of bias assessed by ROBINS-I, and 9 studies were rated as some concerns risk of bias assessed by RoB-2.
